# Integrated CO_2_ Capture and Conversion to Formate with a Molecular Platinum Bis(diphosphine) Electrocatalyst

**DOI:** 10.1021/jacsau.5c00801

**Published:** 2025-10-23

**Authors:** Ciara N. Gillis, Hunter Pauker, R. Dominic Ross, Christopher Hahn, Robert J. Nielsen, Jenny Y. Yang

**Affiliations:** † Department of Chemistry, 8788 University of California, Irvine, Irvine, California 92697, United States; ‡ Department of Chemical and Biological Engineering, University of California, Irvine, Irvine, California 92697, United States; § Materials Science Division, 4578 Lawrence Livermore National Laboratory, Livermore, California 94550, United States

**Keywords:** reactive capture, carbon dioxide, capture, electrocatalysis, sorbent, formate

## Abstract

Carbon dioxide is a potentially valuable feedstock for carbon-based fuels or commodities but is only available in dilute streams. Many studies have focused on either the capture and concentration of CO_2_ or the reduction of pure CO_2_ streams. The direct reduction of sorbent-captured CO_2_ in an integrated process would skip the energy-intensive CO_2_ concentration and sorbent regeneration step. Herein, we report the electrocatalytic reduction of 1,3-bis­(2,6-diisopropylphenyl)­imidazolium-2-carboxylate (IPr·CO_2_), which forms quantitatively from the reaction of sorbent 1,3-bis­(2,6-diisopropylphenyl)­imidazol-2-ylidene (IPr) with 10% and 0.04% CO_2_ streams, by catalyst [Pt­(dmpe)_2_]­(PF_6_)_2_ (dmpe = 1,2-bis­(dimethylphosphino)­ethane) to formate with >70% Faradaic efficiencies. Unexpectedly, experimental studies indicate that the proton source phenol facilitates rapid decarboxylation of IPr·CO_2_ to release CO_2_, which is the substrate for reduction. Kinetic studies determined the rate of hydride transfer from a catalytic intermediate [HPt­(dmpe)_2_]­(PF_6_) to form the C–H bond in formate to be 0.22 M^–1^s^–1^. Further details on the mechanism, transition state energy, and structure for hydride transfer to CO_2_, a common step in CO_2_ reduction, were explored using computational methods.

## Graphical abstract

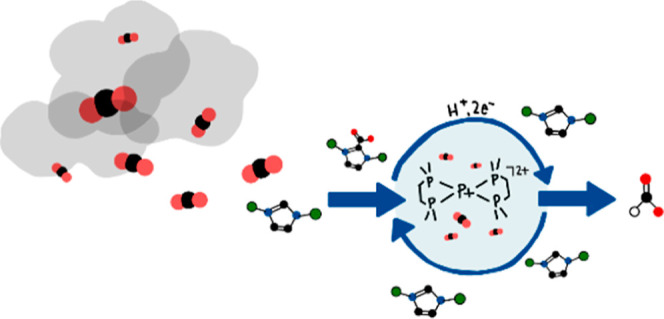

## Introduction

Carbon dioxide (CO_2_) capture and utilization (CCU) is an important component to closing the anthropogenic carbon cycle.^1^ Carbon capture is most commonly investigated independently from utilization, with the former focused on concentrating dilute streams of CO_2_, and the latter focused on valorizing these purified streams. However, the direct use of dilute CO_2_ streams without a separate purification step, or integrated CO_2_ capture and conversion, is drawing increased attention as it can reduce both capital and operational costs for CCU.^2^ The most common motif for integrated CCU involves the use of a sorbent that binds to CO_2_ from dilute concentrations. The resulting sorbed-CO_2_ species is directly reduced as the substrate, generating the carbon-based product and regenerating the sorbent.

However, the introduction of a sorbent into an integrated catalytic system presents new challenges. Because CO_2_ is an electrophile, sorbents are typically nucleophilic. These nucleophiles can competitively bind to the catalytic active sites, resulting in catalyst poisoning or corrosion.^3^ Amines have been the most commonly studied sorbent in integrated capture and electrocatalytic conversion systems, as they are currently used in point source CO_2_ capture and concentration. However, amines form an equivalent of ammonium upon capture,^4^ which can promote hydrogen evolution and loss of Faradaic efficiency for carbon-based products.^3,5,6^ Additionally, most sorbed-CO_2_ species result in anionic species, such as carbamates or carbonates from amines and alkoxides, respectively, that are inhibited in their approach to negatively charged or polarized catalyst sites.^3,7–9^ The sorbent and corresponding sorbed-CO_2_ species must also be stable under reducing conditions to avoid deleterious redox processes in electrocatalytic systems.

Based on these considerations and the challenges that have been reported in the literature, a successful integrated CO_2_ capture and conversion system must ensure sorbent and catalyst compatibility. Additionally, the use of a sorbent that does not require the generation of an acidic species such as an ammonium cation or form an anionic sorbed CO_2_ is beneficial for improving reactivity and selectivity. To this end, we have been exploring the use of an N-heterocyclic carbene (NHC) as a sorbent, which forms a zwitterion upon reaction with CO_2_. NHCs have been used in the organic literature for both capture and utilization of CO_2_ using stoichiometric reactants, although they have been minimally explored in electrocatalytic systems.^10,11^ Our NHC of choice is shown in Scheme 1, 1,3-bis­(2,6-diisopropylphenyl)­imidazol-2-ylidene (IPr), which becomes 1,3-bis­(2,6-diisopropylphenyl)­imidazolium-2-carboxylate (IPr·CO_2_) upon sorption of CO_2_. Both IPr and IPr·CO_2_ are commercially available and soluble in most organic solvents, facilitating our studies. Additionally, IPr has a calculated CO_2_ binding constant of >10^3^ in organic solvents.

**Scheme 1:**
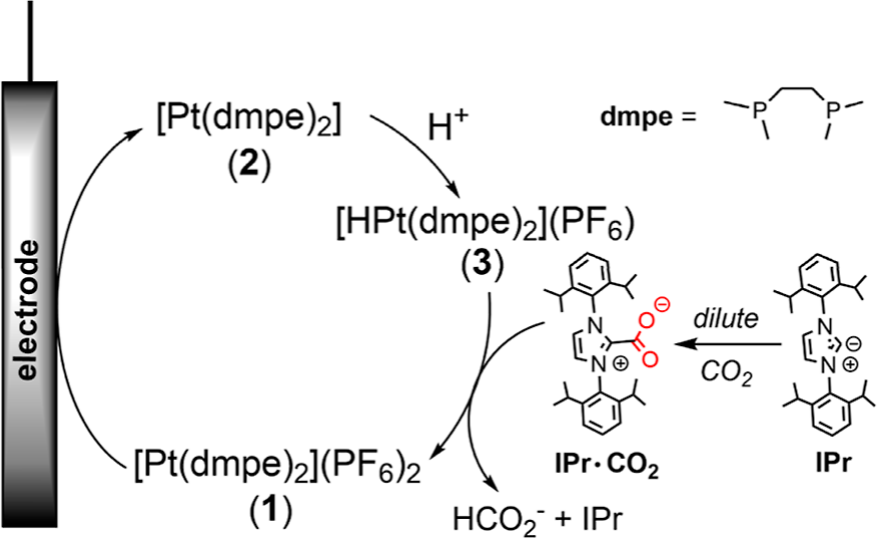
Integrated CCU Scheme with IPr Sorbent Capturing CO_2_ from Dilute Streams to Generate IPr·CO_2_

Our investigation focused on the catalyst [Pt­(dmpe)_2_]­(PF_6_)_2_ (**1**) where dmpe = 1,2-bis­(dimethylphosphino)­ethane (Scheme 1), which we have previously studied for the selective reduction of pure CO_2_ to formate (HCO_2_^–^).^12,13^ In our prior studies, we identified the use of phenol as a proton source to generate the hydride without the concomitant generation of hydrogen, leading to high product selectivity for CO_2_ reduction to formate.^12,13^ In addition to the catalyst resting state, intermediates in the proposed electrocatalytic cycles Pt­(dmpe)_2_ (**2**) and [HPt­(dmpe)_2_]­(PF_6_) (**3**) were independently isolated and characterized, enabling the independent study of each proposed catalytic step with the sorbent.

In this study, we demonstrate that [Pt­(dmpe)_2_]­(PF_6_)_2_ (**1**) is a selective catalyst for the reduction of with >70% Faradaic efficiency (FE) to HCO_2_^–^. IPr·CO_2_ can be added directly as the substrate or generated in situ from dilute (10% and 0.04%) CO_2_ streams. We found that the acid phenol plays a dual role in catalysis. While it serves its intended purpose as a proton source, experimental evidence indicates that it also facilitates free CO_2_ release from IPr·CO_2_. We further investigated the hydride transfer step to CO_2_ that is key to C–H bond formation.

## Results and Discussion

### Synthesis

The catalyst [Pt­(dmpe)_2_]­(PF_6_)_2_ (**1**) and proposed intermediates Pt­(dmpe)_2_ (**2**) and [HPt­(dmpe)_2_]­(PF_6_) (**3**) were all synthesized according to literature procedures.^14,15^ All species are diamagnetic, with previously reported diagnostic ^1^H and ^31^P­{^1^H} NMR spectroscopy resonances.

### Electrocatalytic Studies

Cyclic voltammetry (CV) of the catalyst [Pt­(dmpe)_2_]­(PF_6_)_2_ (**1**) in the absence of a substrate exhibits a two-electron quasireversible reduction event with a half-wave potential (E_1/2_) of −1.73 V vs Fe­(C_5_H_5_)_2_^+/0^ (Figure 1 top, black trace).^12^ As described in our prior studies, addition of 40 mM phenol results in a loss of reversibility from protonation of [Pt­(dmpe)_2_] (**2**) to form [HPt­(dmpe)_2_]­(PF_6_) (**3**) (Figure 1 top, green trace).^14,15^ The trace does not change appreciably with the addition of IPr·CO_2_ (Figure 1 top, blue trace).

**Figure 1:**
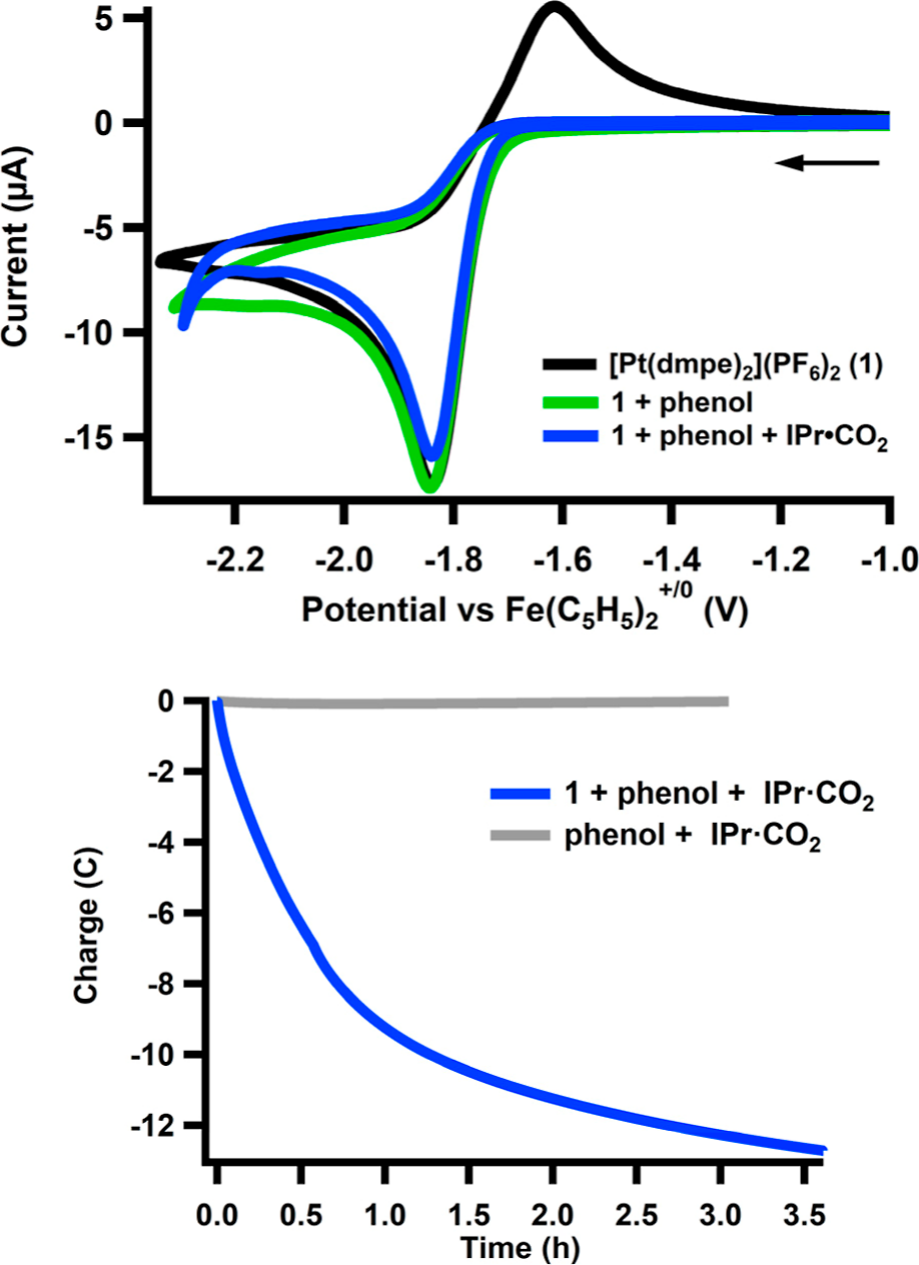
(Top) Cyclic voltammetry of [Pt­(dmpe)_2_]­(PF_6_)_2_ (**1**) (1 mM) only (black trace), after addition of phenol (40 mM) (green trace), and with further addition of IPr·CO_2_ (40 mM) (blue trace) in CH_3_CN (0.1 M TBAPF_6_), at scan rates of 100 mV/s. (Bottom) Representative charge versus time trace from a controlled potential electrolysis (CPE). Conditions for the blue trace where the catalyst is present: 1 mM [Pt­(dmpe)_2_]­(PF_6_)_2_ (**1**), 40 mM phenol, 40 mM IPr·CO_2_, 0.1 M TBAPF_6_ in CH_3_CN. Conditions for the gray trace in the absence of a catalyst: 40 mM IPr·CO_2_, 40 mM phenol, 0.1 M TBAPF_6_ in CH_3_CN.

Controlled potential electrolysis (CPE) at −2.0 V vs Fe­(C_5_H_5_)_2_^+/0^ with [Pt­(dmpe)_2_]­(PF_6_)_2_ (**1**) (1 mM), IPr·CO_2_ (40 mM), and phenol (40 mM) in CH_3_CN under 1 atm N_2_ for 4 h passed 13 ± 4 Coulombs, or an average of 13 equiv of electrons per catalyst (Figure 1 bottom, blue trace). A current versus time trace is shown in Figure S3. Formate was the only product observed in solution by ^1^H NMR spectroscopy and was quantified using an internal standard (Figure S21), to give a Faradaic efficiency (FE) of 82 ± 12%, corresponding to 5.3 equiv per catalyst and 5 turnovers (Table 1). Hydrogen was detected in the headspace using gas chromatography (GC), with an FE of 3 ± 1%; no CO was detected. After electrolysis, ^31^P­{^1^H} NMR spectroscopy confirmed the quantitative retention of the catalyst relative to a standard containing 1 mM [Pt­(dmpe)_2_]­(PF_6_)_2_ (**1**) and 100 mM TBAPF_6_ (tetrabutylammonium hexafluorophosphate) (Figure S22). We note that charge accumulation slows after about one hour. We performed a separate experiment where we added additional substrate after 2 h and found the current increased again. The FE for HCO_2_^*–*^ after this experiment was 90%. These results indicate that the loss of activity is due to substrate consumption (Figure S4). In the absence of the catalyst, an equivalent CPE passed only 22 mC (Figure 1 bottom, gray trace). The only product detected by GC when no catalyst is present is H_2_. No formate- or other carbon-based products were observed in the ^1^H NMR spectrum (Figure S23). As the FE values for HCO_2_^–^ and H_2_ together are not 100%, there is some unaccounted charge. To investigate other potential reductive reactions, we performed a CPE with 1 mM [Pt­(dmpe)_2_]­(PF_6_)_2_ (**1**), 40 mM phenol, and 40 mM of the sorbent IPr; 4C of charge passed over four hours, indicating a modest background reaction (Figure S5).

**Table 1:** Faradaic Efficiencies for Electrolysis with 1 mM [Pt­(dmpe)_2_]­(PF_6_)_2_ (**1**), 40 mM IPr·CO_2_, 40 mM Phenol, and 0.1 M TBAPF_6_ in CH_3_CN^a^

substrate	FE HCO_2_^–^ (%)	FE H_2_ (%)	TON (%)
IPr·CO_2_	82(12)	3(1)	5.0(1.7)
IPr, 10% CO_2_	72	1	6.8
IPr, 0.04% CO_2_	72	1	1.3

a The first entry used commercial IPr·CO_2_, while the second and third entry represent generation of IPr·CO_2_ in situ from IPr (40 mM) from 10% and 0.04% CO_2_ in N_2_ over the course of 30 min and 3 h, respectively. All CPEs were performed at −2.0 V vs Fe­(C_5_H_5_)_2_^+/0^ for 4 h.

A controlled potential electrolysis with phenol-*d*_6_ confirmed that the proton in formate arises from phenol. A resonance corresponding to DCO_2_^–^ is in the ^2^H NMR spectrum (Figure S24). No deuterium was incorporated into the other protons on the substrate or catalyst.

We also tested the in situ generation of IPr·CO_2_ using pure CO_2_ as well as dilute streams. Isotopically labeled ^13^CO_2_ was used to generate IPr·^13^CO_2_ prior to electrolysis. Formate (H^13^CO_2_^–^) was detected by both ^1^H and ^13^C NMR spectroscopy (Figures S25 and S26). To more closely mimic capture and utilization of flue gas, a dilute stream of 10% CO_2_ (90% N_2_) was used to generate IPr·CO_2_ (40 mM) in 0.1 M TBAPF_6_ in CH_3_CN, followed by the addition of [Pt­(dmpe)_2_]­(PF_6_)_2_ (**1**) and then phenol. The experiment was performed at the same potential (−2.0 V vs Fe­(C_5_H_5_)_2_^+/0^) under a N_2_ atmosphere, and Faradaic efficiencies (FEs) of 72% formate and 1% H_2_ were achieved (Table 1). Equivalent FEs were observed with 0.04% CO_2_ (99.96% N_2_) to represent capture from an air-relevant concentration. Thus, the generation of IPr·CO_2_ using dilute CO_2_ does not significantly interfere with the reduction to HCO_2_^–^.

### Mechanistic Studies

#### [Pt­(dmpe)_2_]­(PF_6_)_2_ and Pt­(dmpe)_2_ Reactivity with IPr·CO_2_ and IPr

As noted in the Introduction, nucleophilic sorbents have the potential to interfere with and potentially poison catalyst active sites. To ensure the sorbent (IPr) and sorbed-CO_2_ species (IPr·CO_2_) do not interact unfavorably with catalytic species [Pt­(dmpe)_2_]­(PF_6_)_2_ (**1**) or [Pt­(dmpe)_2_] (**2**), we examined their reactivity independently.

Independently synthesized [Pt­(dmpe)_2_]­(PF_6_)_2_ (**1**) (Figure S12) has a resonance in the ^31^P­{^1^H} NMR spectrum at 33.7 ppm. Spectra with an added equimolar amount of IPr·CO_2_ (Figure S13) and IPr (Figure S15) show no changes, demonstrating that the sorbent and sorbed CO_2_ do not interact with the catalyst resting state.

[Pt­(dmpe)_2_] (**2**) was also independently synthesized and tested for its reactivity with IPr and IPr·CO_2_. [Pt­(dmpe)_2_] (**2**) has a resonance in the ^31^P­{^1^H} NMR spectra located at −12.0 ppm (Figure S17). The addition of equimolar IPr results in no changes in the ^31^P­{^1^H} NMR spectrum (Figure S18). The lack of reactivity with either [Pt­(dmpe)_2_]­(PF_6_)_2_ (**1**) or [Pt­(dmpe)_2_] (**2**) and IPr is also evident from cyclic voltammetry. No changes to the cyclic voltammetry of [Pt­(dmpe)_2_]­(PF_6_)_2_ (**1**) occur after the addition of IPr (Figure S10).

However, the addition of IPr·CO_2_ to the reduced compound [Pt­(dmpe)_2_] (**2**) results in a new resonance at 26.8 ppm in the ^31^P­{^1^H} NMR spectra (Figure S19). Scan-rate-dependent studies demonstrate a loss of anodic current with slower scan rates (Figure S6). This scan-rate-dependent behavior indicates IPr·CO_2_ interacts with the reduced catalyst [Pt­(dmpe)_2_] (**2**) in an EEC reaction, where EE represents the two-electron reduction followed by C, a chemical step, similar to what is observed when phenol is added. Scan-rate-dependent studies with [Pt­(dmpe)_2_]­(PF_6_)_2_ (**1**) and IPr·CO_2_ were used to obtain the rate of reaction of [Pt­(dmpe)_2_] (**2**) and IPr·CO_2_. At higher scan rates, the anodic wave returns and has a scan-rate-dependent peak current. The scan-rate-dependent ratio of the peak anodic and cathodic currents was compared to the total mV traversed to determine the half-life of the reaction. An observed rate (*k*_obs_(IPr·CO_2_)) of 0.41 ± 0.04 s^–1^ was determined with 1 mM Pt complex and 20 mM IPr·CO_2_ (Figures S6 and S7).^10,16–20^

We previously determined the observed rate of protonation (*k*_obs_(H^+^)) of [Pt­(dmpe)_2_] (**2**) by phenol under catalytic conditions to be greater than 10^5^ s^–1^, which is orders of magnitude faster than the observed rate of its reaction with IPr·CO_2_. As a result, even though reactivity of IPr·CO_2_ with [Pt­(dmpe)_2_] (**2**) is possible, hydride formation is favored under electrocatalytic conditions.^15^

#### IPr·CO_2_ and IPr Reactivity with Phenol

In the course of our electrocatalytic studies, we noted the presence of CO_2_ in our postelectrolysis solutions, even when the experiment only used IPr·CO_2_ under 1 atm of N_2_ and was never exposed to CO_2_ gas (Figure S27). The presence of free CO_2_ was unexpected. The equilibrium binding constant for CO_2_ is expected to be >10^3^ since IPr·CO_2_ is formed quantitatively with 0.04% CO_2_ streams with IPr (Figures S28 and S29). Consistent with this experimental result, the calculated free energy for CO_2_ binding to IPr is 5.1 and 8.3 kcal/mol in DMSO^13^ and CH_3_CN,^22^ respectively. Thus, we would expect the release of free CO_2_ to be unfavorable.

To investigate the possibility of the release of CO_2_ from IPr·CO_2_, the headspace of IPr·CO_2_ in CH_3_CN was monitored for CO_2_ over time (Figure 2, top). Before 160 min, no CO_2_ levels above background (<100 ppm) were detected. However, at 160 min, an equimolar amount of phenol was added, showing an immediate increase in CO_2_ concentration (>2000 ppm) in the headspace. The same experiment was also conducted where the headspace gas was monitored immediately upon the addition of an equimolar amount of phenol (Figure 2, bottom). In the initial measurement, CO_2_ is present at concentrations >2000 ppm. The continuous sweep of inert gas that enables the headspace measurement results in a lower concentration of CO_2_ over time. These experiments demonstrate that IPr·CO_2_ alone in CH_3_CN is stable to CO_2_ release, which is consistent with its use for CO_2_ capture from dilute streams. However, the addition of the acid source phenol results in a rapid release of free CO_2_. We attempted to obtain rate information about the release of CO_2_ from IPr·CO_2_ upon addition of phenol using UV–visible spectroscopy (Figure S37). One equivalent of phenol was added to a 15 mM IPr·CO_2_ solution in CH_3_CN. Upon completion of the first scan at 7 s, the feature associated with IPr·CO_2_ was no longer present, indicating rapid completion of the reaction and formation of HIPr.

**Figure 2:**
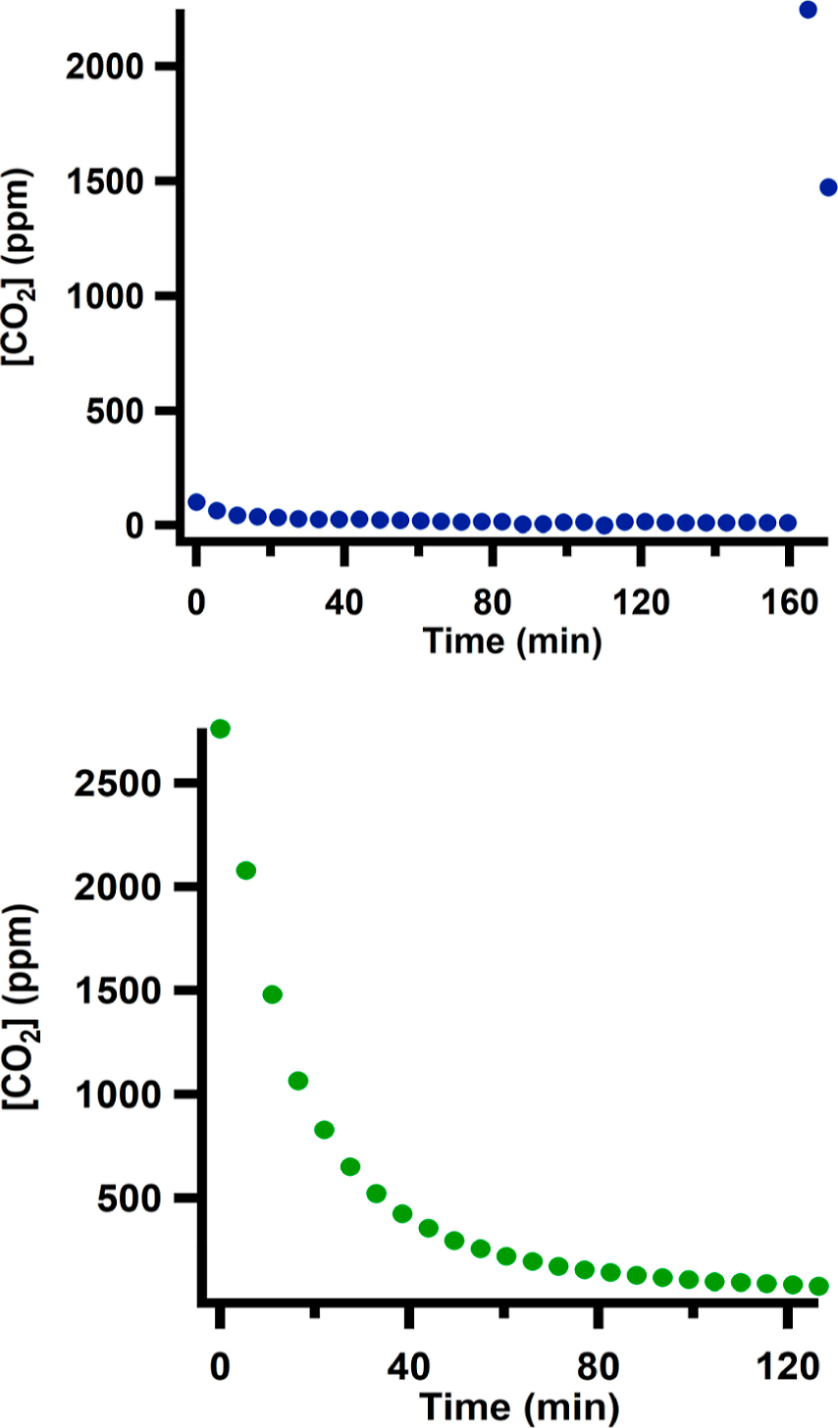
Headspace CO_2_ concentration detected by online gas chromatography, with data collected in 30 s intervals. (Top) 40 mM IPr·CO_2_ in CH_3_CN, phenol was added after 160 min. (Bottom) At *t* = 0, phenol (40 mM) was added to IPr·CO_2_ (40 mM) in CH_3_CN.

Release of CO_2_ from IPr·CO_2_ would require an interaction between phenol and IPr that is more favorable than CO_2_ binding. To probe this interaction, excess phenol was added to a solution of IPr in CH_3_CN. Single crystals were isolated from this reaction. Analysis by X-ray diffraction revealed a protonated IPr with hydrogen-bonding interactions between a phenoxide and two additional equivalents of phenol (Figure 3). These types of hydrogen-bonding interactions between moderately acidic protons and carbenes have previously been described between carbenes and alcohols and amines.^23–25^ The ^1^H NMR spectrum upon titration of phenol to IPr shows that the phenol protons in the aromatic region shifted more downfield, which is indicative of hydrogen bonding (Figure S31). Titration of phenol into a solution of IPr·CO_2_ in CD_3_CN also displays similar shifts in proton resonances (Figure S32).

**Figure 3:**
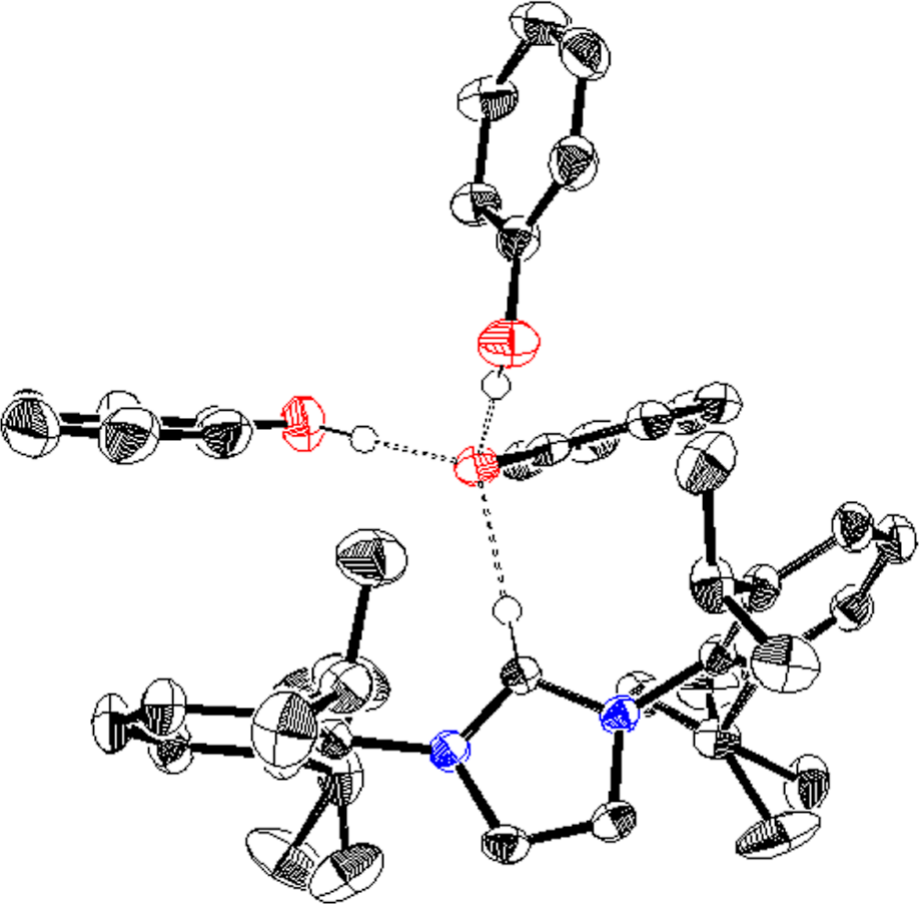
Solid-state structure resulting from the reaction of IPr and phenol. Hydrogen atoms that do not participate in hydrogen bonding have been omitted for clarity. The protons shown were located from the difference map. Thermal ellipsoids are represented at 60% probability.

Further evidence that IPr·CO_2_ is not the direct substrate for reduction stems from stoichiometric studies of [HPt­(dmpe)_2_]­(PF_6_) (**3**) with IPr·CO_2_. The reaction between [HPt­(dmpe)_2_]­(PF_6_) (**3**) and 20 equiv of IPr·CO_2_ was monitored by ^31^P­{^1^H} NMR spectroscopy (Figure S30). It was only after ∼28 h that hydride transfer to form HCO_2_^–^ and [Pt­(dmpe)_2_]­(PF_6_)_2_ (**1**) was 94% complete, indicating that in the absence of phenol, formate production is slower. Moreover, in the presence of various concentrations of the transformation of [HPt­(dmpe)_2_]­(PF_6_) (**3**) into [Pt­(dmpe)_2_]­(PF_6_)_2_ (**1**) has a second-order rate constant of 1.8 × 10^–3^ M^–1^ s^–1^ (Figure S33).

#### Hydride Transfer to CO_2_

The prior experimental data indicate that the addition of phenol results in rapid CO_2_ release from IPr·CO_2_, whereas hydride transfer to IPr·CO_2_ is slow (1.8 × 10^–3^ M^–1^ s^–1^). These results indicate that CO_2_ is the likely substrate for reduction by [HPt­(dmpe)_2_]­(PF_6_) (**3**) to form HCO_2_^–^. Although we had previously described the reduction of CO_2_ to formate (HCO_2_^–^) by this catalyst via hydride transfer from the intermediate [HPt­(dmpe)_2_]­(PF_6_) (**3**), we had not previously investigated the kinetic parameters or mechanism for this step.

The rate of hydride transfer to CO_2_ was determined by using UV–visible spectroscopy. [HPt­(dmpe)_2_]­(PF_6_) (**3**) was treated with varying concentrations of CO_2_ in nitrogen. The decay of the [HPt­(dmpe)_2_]­(PF_6_) (**3**) absorption band at 332 nm was monitored over time to give *k*_obs_ values at each CO_2_ concentration (Tables S3, S4 and Figure S35). The final spectrum corresponds to the quantitative formation of [Pt­(dmpe)_2_]­(PF_6_)_2_ (**1**), which is characterized by a new absorbance band at 296 nm (Figure S34). The relationship between *k*_obs_ and the concentration of CO_2_ was used to find the second-order rate constant of 0.22 M^–1^ s^–1^ (Figure S36). The rate of insertion of CO_2_ into various metal hydride bonds to form formate has been investigated in several cases, most notably by Hazari and co-workers. Rate constants for these measurements range from 10^–4^ to 10^2^ M^–1^ s^–1^.^26–31^ The rate of reaction of CO_2_ with [HPt­(dmpe)_2_]­(PF_6_) (**3**) falls within the range observed for metal hydrides.

#### Computational Studies

DFT modeling was used to explore the speciation and reaction routes available to IPr·CO_2_ and [HPt­(dmpe)_2_]­(PF_6_) (**3**) under operating conditions. Structures were optimized in the SMD solvation model^32^ representing CH_3_CN with the TPSS0-D3 functional^33–37^ and augmented def2-TZVP^38,39^ basis, using Orca 5.0.4.^40,41^ A pH of 29 (the p*K*_a_ of phenol) and a potential of −1.8 V vs Fe­(C_5_H_5_)_2_^+/0^, or −1.16 V vs SHE (adopting values of 0.24 V for SCE vs SHE and 0.40 V for Fe­(C_5_H_5_)_2_^+/0^ in CH_3_CN vs SCE^42^), were used in computing relative free energies (additional details can be found in the Supporting Information). Figure 4 contains free energies of species that might result from reactions of IPr·CO_2_ and phenol at the electrode. One-electron reduction of IPr·CO_2_ to **A** (Figure 4) is endergonic by 29.3 kcal/mol, which is consistent with the measured reduction potential at −2.8 V vs Fe­(C_5_H_5_)_2_^+/0^ (Figure S9). Protonation to form **B** and subsequent reduction to form **C** or **C′** are also unfavorable under these conditions and not observed experimentally. Addition of two electrons and one or two protons (**H** or **I**) to IPr·CO_2_ is less endergonic; however, we found no low-barrier pathway for forming the C–H bond of formate proceeding from these intermediates.

**Figure 4:**
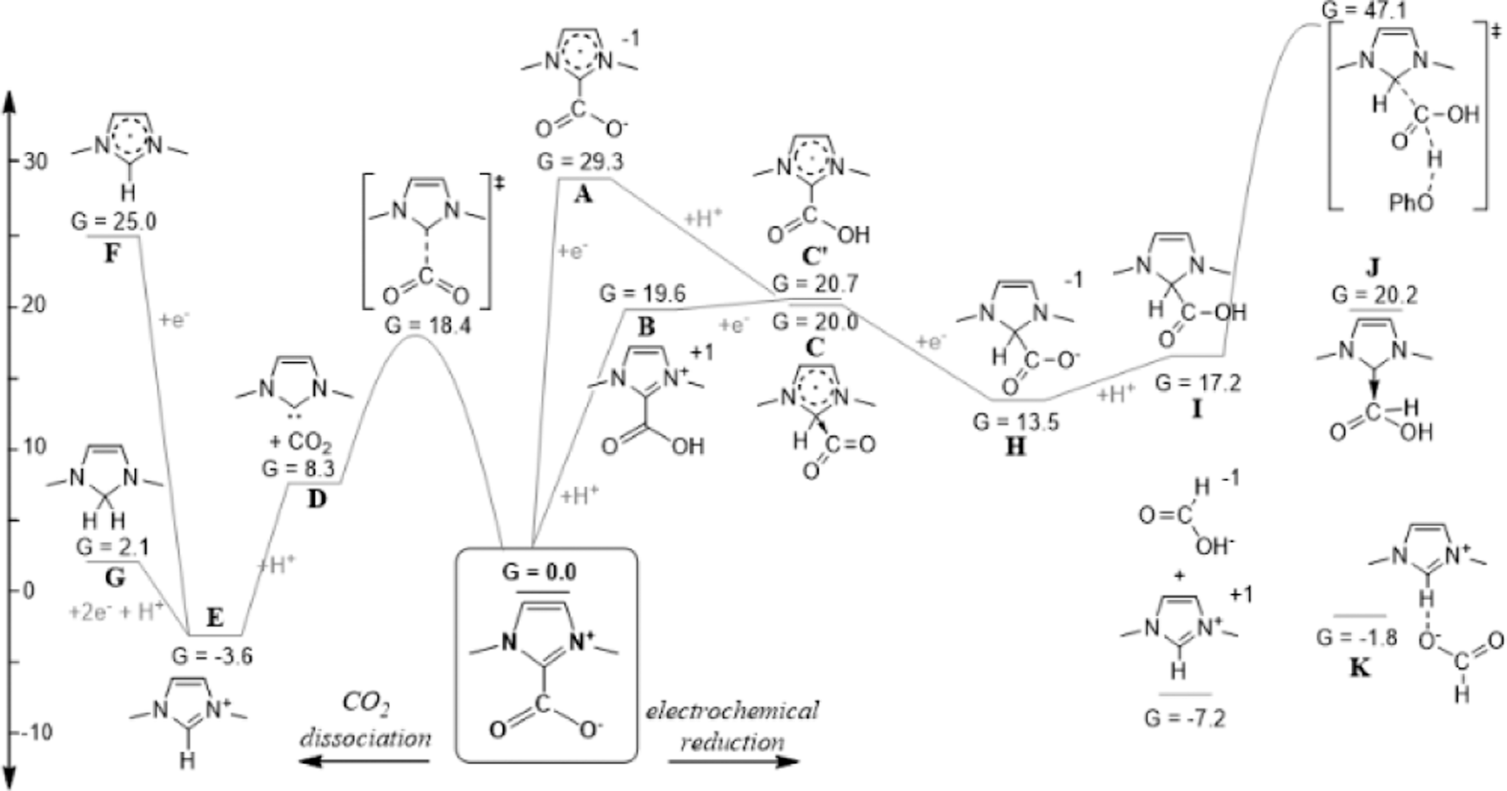
Calculated standard free energies of formation in CH_3_CN at pH 29 and −1.8 V vs Fe­(C_5_H_5_)_2_^+/0^ (kcal/mol). 2,6-Diisopropylphenyl groups of IPr are omitted for clarity.

The C–C bond dissociation free energy in IPr·CO_2_ (at 1 atm of CO_2_) is 8.3 kcal/mol, with a dissociation barrier of 18.4 kcal/mol. Protonation of the free carbene (**D** to **E**) at pH 29 is calculated to be exergonic 11.9 kcal/mol. We therefore infer that the role of phenol is to rapidly protonate IPr and render the dissociation of CO_2_ effectively exergonic by 3.6 kcal/mol, which is consistent with our experimental evidence of rapid release of CO_2_ with phenol. Further reduction of the imidazolium by one electron (**F**) or a hydride ion (**G**) is endergonic at these potentials.^40^ As **G** is only slightly endergonic, we examined our postelectrolysis solution for it by ^1^H NMR spectroscopy^43^ but did not find any evidence of its formation.

Generation of formate was computed to be exergonic by 7.2 kcal/mol under these conditions. Hydrogen-bonded complexes between imidazolium and formate (**H**) or phenoxide, as well as HCO_2_^–^(TBA^+^), were predicted to be less favorable than free formate and imidazolium.

We sought transition states in which hydride is transferred from [HPt­(dmpe)_2_]­(PF_6_) (**3**) to the CO_2_ carbon of IPr·CO_2_ (Figure 5). Steric interference between the dmpe ligands and the isopropyl groups of IPr·CO_2_ was apparent when constructing models, so ab initio molecular dynamics (using a faster BP86 ^44,45^ /def2-SVP^38^ protocol) was used to anneal structures with Pt···H and H···C constraints between 50 and 700 K for a few picoseconds. Subsequent eigenvector following led to saddle point **L**, in which the IPr···CO_2_ bond is completely dissociated. A proton was added to the CO_2_ unit of **L** to render it more electrophilic, resulting in the concerted transition state **M**. However, the activation barriers posed by **L** and **M** are too high to be responsible for the experimentally observed rate of formate formation from [HPt­(dmpe)_2_]­(PF_6_) (**3**) and IPr·CO_2_. These barriers are a little higher than expected from our experimentally measured rate of 1.8 × 10^–3^ M^–1^ s^–1^, but it is possible that small amounts of adventitious water from the solvent or electrolyte may contribute to accelerating the reaction. Ultimately, the only low-barrier path identified was the simple transfer of a hydride from [HPt­(dmpe)_2_]­(PF_6_) (**3**) to dissociated CO_2_ (**N**), which is consistent with our conclusion that free CO_2_ is generated under catalytic conditions and is the substrate. From free CO_2_, **N** poses a calculated standard free-energy barrier of 17.5 kcal/mol (Δ*S* ^‡^ = −43.5 e.u. and Δ*H* ^‡^ = 4.5 kcal/mol). Due to the early character of the transition state (a C···H distance of 1.75A), an H/D kinetic isotope of only 1.3 is predicted for this step. Transition state **L** is essentially the geometry of **N** with IPr present.

**Figure 5:**
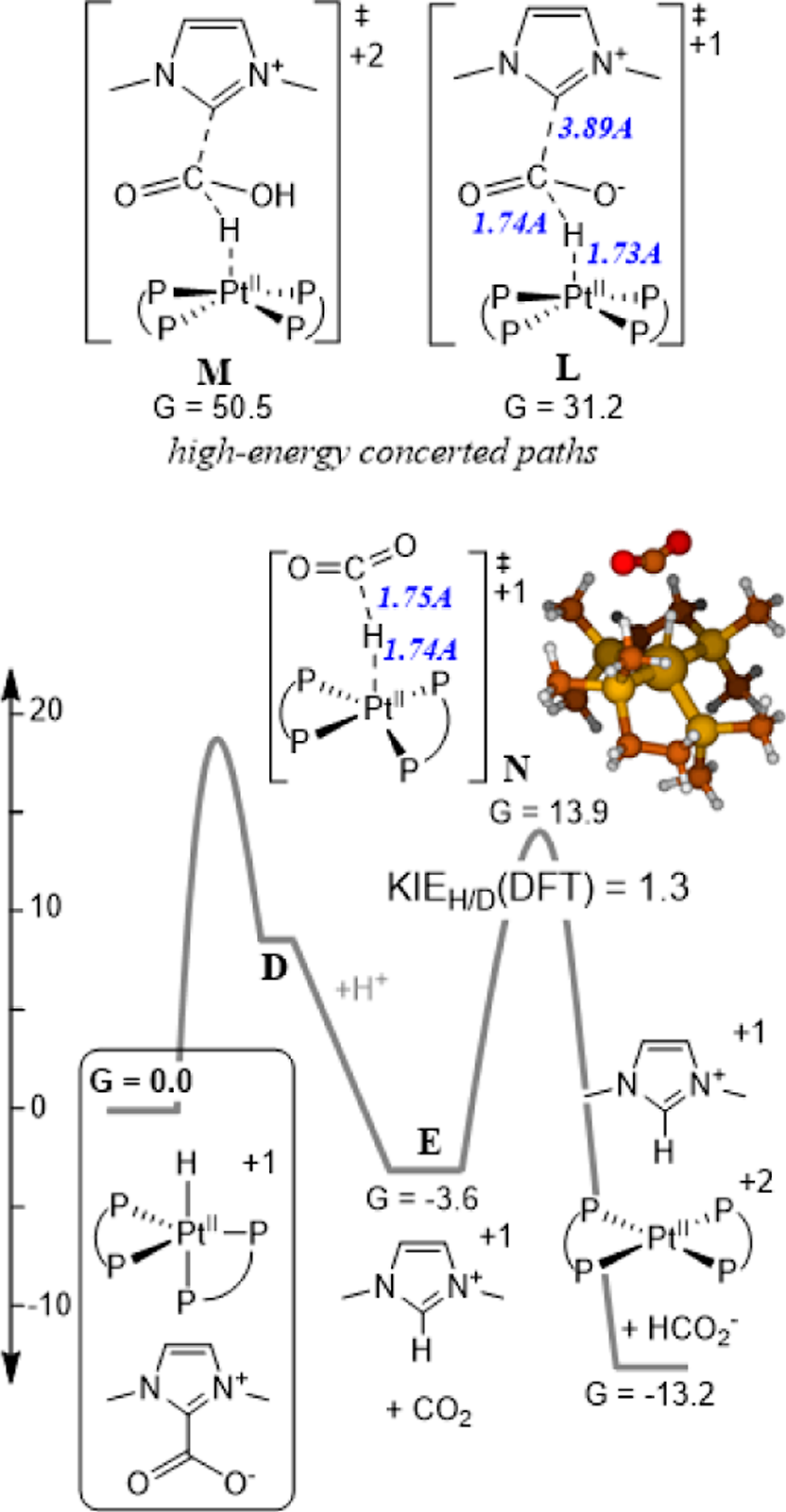
Calculated standard free energies of formation in CH_3_CN at pH 29 and −1.8 V vs Fe­(C_5_H_5_)_2_^+/0^ (kcal/mol), with bond lengths in Angstroms in blue. 2,6-Diisopropylphenyl groups of IPr are omitted for clarity.

#### Impact of the Sorbent

The facile release of CO_2_ from IPr·CO_2_ led to an investigation on the role of the sorbent in facilitating the CO_2_ reduction of dilute streams. CPE studies in the absence of sorbent were run under dilute CO_2_ conditions to examine the impact of IPr on concentrating CO_2_ in solution. The conditions of 1 mM [Pt­(dmpe)_2_]­(PF_6_)_2_ (**1**) and 40 mM phenol were maintained for these experiments. As noted in Table 1, the use of IPr to capture CO_2_ from a 0.04% stream leads to >70% Faradaic efficiency for HCO_2_^–^. In contrast, CPE under 0.04% CO_2_ with no IPr led to no detectable formate. However, CPE with 10% CO_2_ without IPr leads to a similar Faradaic efficiency of HCO_2_^–^ (70%) compared to when IPr is used to capture the CO_2_. We note that 10% CO_2_ is expected to result in a CO_2_ concentration of about 28 mM, which is close to the experimental IPr·CO_2_ concentration of 40 mM. Conversely, 0.04% CO_2_ will result in a concentration of about 0.11 mM. From these experiments, the sorbent IPr plays an important role in capturing and concentrating CO_2_ at dilute concentrations (0.04%) for further reduction. However, it appears to have less impact when the sorbent is at similar concentrations to the dilute stream, which is observed with 10% CO_2_.

## Conclusion

An electrocatalyst with selective activity toward the reduction of CO_2_ to formate was studied with a carbene-captured CO_2_ substrate to determine its effectiveness toward integrated CO_2_ capture and conversion. Electrochemical characterization of the catalyst and substrate, IPr·CO_2_, displayed behavior similar to that of the [Pt­(dmpe)_2_]­(PF_6_)_2_ (**1**) and the pure CO_2_ system, generating high Faradaic efficiencies for formate and minimal hydrogen evolution. The most significant impacts for catalysis are for the most dilute stream (0.04% CO_2_) highlighting the sorbent’s impact in concentrating the substrate. Importantly, the sorbent (IPr) and sorbed-CO_2_ (IPr·CO_2_) species do not interfere with the catalyst.

Despite a high calculated binding constant for CO_2_ to the carbene sorbent (8.1 kcal/mol), we found that the addition of phenol prompts the rapid release of CO_2_. Additional experimental and computational investigations indicate that CO_2_ release is driven by hydrogen bonding between the phenol and sorbent. These results demonstrate that the substrate during reduction is likely free CO_2_. The reduction of CO_2_ by [HPt­(dmpe)_2_]­(PF_6_) (**3**) was further investigated experimentally and computationally to determine the rate of the mechanism of C–H bond formation.

These studies describe a new catalytic system using an NHC sorbent and a CO_2_-to-formate electrocatalyst for the integrated capture and conversion of CO_2_ from dilute streams. Furthermore, we demonstrate how phenol can facilitate free-CO_2_ release under electrocatalytic conditions. These findings describe key considerations for the continued development of integrated catalytic CCU systems.

## Supplementary Material



## Notes

General, electrochemical measurements, calculations, NMR spectroscopy, computational methods, and X-ray crystallography (PDF)
