# Supplementary material for: Integrated CO2 Capture and Conversion to Formate with a Molecular Platinum Bis(diphosphine) Electrocatalyst
Source: JACS Au. 2025 Oct 23;5(11):5359–66. doi: 10.1021/jacsau.5c00801 (PMC12648282; doi:10.1021/jacsau.5c00801)
Supplement: Supplementary file 1 [file au5c00801_si_001.pdf]

Supporting Information for

Integrated CO_2 Capture and Conversion to Formate with a Molecular Platinum Bis(diphosphine) Electrocatalyst

Ciara N. Gillis[a], Hunter Pauker[b], R. Dominic Ross[c], Christopher Hahn[c], Robert J. Nielsen[b], Jenny Y. Yang[a*]

[a]*Department of Chemistry, University of California, Irvine, Irvine, California, 92697, United States*

[b]*Department of Chemical and Biological Engineering, University of California, Irvine, Irvine, California, 92697, United States*

[b]*Materials Science Division, Lawrence Livermore National Laboratory, Livermore, California, 94550, United States*

*Corresponding author: j.yang@uci.edu

Table of Contents

General Considerations. 2

Electrochemical Measurements 2

Calculations 3

Cyclic Voltammetry 5

NMR Spectroscopy 11

Kinetic Experiments 32

Crystal Structure Data 35

Computational Details 37

References 38

General Considerations. All manipulations were performed in a N_2 filled glovebox (Vacuum Atmospheres OMNI-lab) or through Schlenk line methods. $^{31}P\{^1H\}$, ^1H, and ^{13}C nuclear magnetic resonance (NMR) spectra were collected at room temperature on Bruker AVANCE600 MHz and AV400 MHz spectrometers. Spectra were analyzed using MestRe Nova 6.0.2-5475 software. Post-controlled potential electrolysis spectra were referenced to CD_3CN at 1.94 ppm. A trimethoxybenzene internal reference was used for formate quantification. A triphenylphosphine oxide internal reference was used for $^{31}P\{^1H\}$ NMR spectra. Ultraviolet-visible (UV-vis) spectra were collected using an Agilent Technologies Cary 60 UV-vis spectrometer in a quartz cuvette with a 1 cm path length. Chemical reagents were received from commercial sources without further purification. Nondeuterated organic solvents were degassed by sparging with argon and then dried by passage through an alumina column under argon pressure on a solvent drying system (JC Meyer Solvent Systems) and stored over 3 Å molecular sieves under a N_2 atmosphere. Deuterated solvents were purchased from Cambridge Isotope Laboratories, degassed by freeze-pump-thaw methods, and stored over 3 Å molecular sieves under a N_2 atmosphere. Tetrabutylammonium hexafluorophosphate ($TBAPF_6$) was recrystallized from ethanol three times, dried under vacuum, and stored under a N_2 atmosphere. $[Pt(dmpe)_2](PF_6)_2$ (**1**), $Pt(dmpe)_2$ (**2**), and $[HPt(dmpe)_2](PF_6)$ (**3**) were synthesized according to literature procedure.[1,2]

CO_2 Release from IPr·CO_2

Experimental Details and Procedures. IPr·CO_2 and phenol were added to a round-bottom three-neck flask with a total volume of about 50 mL. The flask was sparged with 10 mL/min of argon for at least 15 minutes while CO_2 background measurements were taken by an on-line gas chromatograph (GC). Background concentrations of CO_2 were <100 ppm. Then, 10 mL of acetonitrile (99.9%, Extra Dry over Molecular Sieve, Thermo Scientific Chemicals) was injected into the flask through a rubber septum so that the resulting concentrations of IPr·CO_2 and phenol were each 40 mM. Samples of the headspace were injected into the GC every 5.5 minutes (5 minute GC run with a 30 second cooldown). A control experiment was conducted with the same method but without the initial addition of phenol.

The GC was an SRI MG#5 with a 6 ft HayeSep D column and a flame ionization detector used for CO_2 separation and detection, respectively. The concentration of CO_2 was determined by calibration with a known mixture containing 1% CO_2.

Electrochemical Measurements

Electrochemical techniques were performed using a Pine Wavedriver 10 bipotentiostat with AfterMath software. All electrochemical experiments were performed in a N_2 filled glovebox.

Cyclic Voltammetry. Cyclic voltammetry (CV) measurements were studied with a 1 mm glassy carbon working electrode, a glassy carbon rod counter electrode, and a silver wire reference electrode. All CV experiments were internally referenced to $Fe(C_5(CH_3)_5)_2^{+/0}$ (Fc*) unless otherwise noted. Scans were further corrected to $Fe(C_5H_5)_2^{+/0}$ ($Fc^{+/0}$) by shifting the CVs an additional 0.505 V cathodically. This correction was determined using $Fc^{+/0}$ and Fc* that were both purified by sublimation. CVs were scanned cathodically in CH_3CN with 0.1 M $TBAPF_6$ and corrected for approximately 80% of the Ohmic drop. Scan rate dependent CVs show linear

dependence for the peak cathodic and anodic currents as a function of the square root of the scan rate (Figures S1 and S2). This correction also matches the reduction potential previously reported by Ceballos et al.[3]

Controlled Potential Electrolysis. Controlled potential electrolysis (CPE) experiments were performed in a custom H-cell with a gas bridge (Adams & Chittenden Scientific Glass Corp). The total cell volume was 28 mL, including 12.5 mL of headspace. The working and counter compartments were separated by a porous glass frit and sealed with GL25 and GL18 caps with silicone/PTFE septa from Ace Glass. The working compartment was filled with 10 mL of 0.1 M $TBAPF_6$ in CH_3CN, a stir bar, the working electrode, and reference electrode. The working electrode was a piece of carbon cloth (~1.5 cm x 4 cm) attached by copper wire. A glass jacketed $Ag^{+/0}$ wire reference electrode in 0.1 M $TBAPF_6$ in CH_3CN separated from the bulk solution by a Vycor frit was used. The counter compartment consisted of 5.5 mL of 0.1 M $TBAPF_6$ in CH_3CN solution, a stir bar, and the counter electrode. The counter electrode was a piece of carbon cloth (~1 cm x 4 cm) attached with copper wire. Excess ferrocene was used in the counter compartment as a sacrificial reductant.

Each CPE was performed at -2.0 V vs. $Fc^{+/0}$ which was approximately 200 mV past the cathodic peak. After electrolysis, a Restek A-2 Luer slip gas-tight syringe was used to sample the headspace of the working compartment. Hydrogen was quantified by an Agilent 7890B gas chromatograph with a HP-PLOT molecular sieve column (19095P-MS6, 30m x 0.530 mm, 25 μm) and TCD detector with an Ar carrier gas. Formate was quantified by adding a 0.5 mL aliquot of the working compartment solution to an NMR tube with 0.1 mL of 18 mM trimethoxybenzene in CD_3CN. A ^1H NMR spectrum was acquired with 8 scans and a delay time of 10 seconds. A $^{31}P\{^1H\}$ NMR spectrum was acquired with 64 scans. A $^{31}P\{^1H\}$ NMR spectra of 1 mM $[Pt(dmpe)_2](PF_6)_2$ (**1**) and 0.1 M $TBAPF_6$ was used as a standard to ensure that there was no decomposition or paramagnetic species post-electrolysis.

Calculations

Product Quantification and Faradaic Efficiency. Formate produced during electrolysis was quantified by taking a 0.5 mL aliquot of the working compartment (WC) solution and 0.1 mL of a 18 mM 1,3,5-trimethoxybenzene solution in CD_3CN as an internal standard. A ^1H NMR spectrum was acquired with 8 scans and a delay time of 10 seconds. The integration of the formyl proton at 8.6 ppm was compared to that of the internal standard and was used to obtain the amount of formate in moles. A sample calculation is below.

$$\frac{[TMB]}{int\ of\ TMB} = \frac{[HCOO^-]}{int\ of\ HCOO^-}$$

$$\frac{3\ mM}{3} = \frac{x}{0.86}$$

$$x = 2.58\ mM\ HCOO^-$$

$$M_{NMR}V_{NMR} = M_{bulk}V_{bulk}$$

$$M_{bulk} = \frac{(2.58\ mM)(0.60\ mL)}{10.0\ mL} = 3.10\ mM\ HCOO^{-}\ in\ WC$$

With 6.235 C passed in a solution and 2 mol e^{-} needed for catalysis,

$$6.235\ C \times \frac{1\ mol\ e^{-}}{96486\ C} \times \frac{1\ mmol}{2\ mol\ e^{-}} = 0.0323\ mmol\ product\ (theoretical)$$

Therefore,

$$\frac{0.0310\ mmol\ HCOO^{-}\ generated}{0.0323\ mmol\ HCOO^{-}\ theoretical} \times 100\% = 95.8\%\ Faradaic\ efficiency$$

H_2 produced during electrolysis was quantified by injecting a 0.1 mL sample into the GC instrument. Integrations were analyzed using a calibration curve to determine the percentage of H_2 in the headspace of the cell. The percentage was then compared to the headspace of the cell to give a quantity of H_2 in mL. The mL amount of H_2 was converted to moles using the ideal gas law and that quantity was compared to the theoretical amount of H_2 produced to find Faradaic efficiency. A sample calculation is below.

0.15% H_2 of 12.5 mL headspace =0.018 mL H_2

$$n = \frac{PV}{RT}$$

$$n = \frac{(1\ atm)(1.8\ \times 10^{-5}\ L)}{(0.082\ \frac{L\ atm}{mol\ K})(298\ K)} = 7.52\ \times 10^{-4}\ mmol\ H_2$$

With 6.235 C passed in a solution and 2 mol e^{-} needed for catalysis,

$$6.235\ C \times \frac{1\ mol\ e^{-}}{96486\ C} \times \frac{1\ mmol}{2\ mol\ e^{-}} = 0.0323\ mmol\ product\ (theoretical)$$

Therefore,

$$\frac{7.52\ \times 10^{-4}\ mmol\ H2\ generated}{0.0323\ mmol\ H2\ theoretical} \times 100\% = 2.33\%\ Faradaic\ efficiency$$

Cyclic Voltammetry

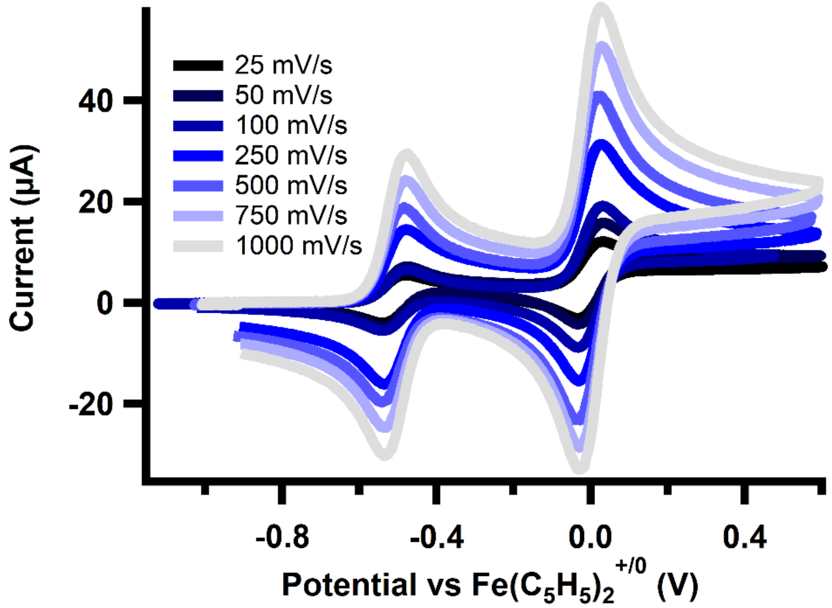

Figure S1. CV of $Fe(C_5H_5)_2^{+/0}$ ($Fc^{+/0}$) and $Fe(C_5(CH_3)_5)_2^{+/0}$ (Fc*) at varying scan rates in CH_3CN (0.1 M $TBAPF_6$). The $E_{1/2}$ value for Fc* is -0.505 V vs $Fc^{+/0}$.

Table S1. Potential peak to peak separation (ΔE_p) of $Fe(C_5H_5)_2^{+/0}$ ($Fc^{+/0}$) and $Fe(C_5(CH_3)_5)_2^{+/0}$ (Fc*) at varying scan rates.

Scan Rate (V/s)	ΔE_p Fc* (V)	ΔE_p Fc (V)
0.025	0.057	0.067
0.05	0.057	0.067
0.10	0.062	0.062
0.25	0.053	0.058
0.50	0.053	0.058
0.75	0.053	0.059
1.0	0.058	0.063

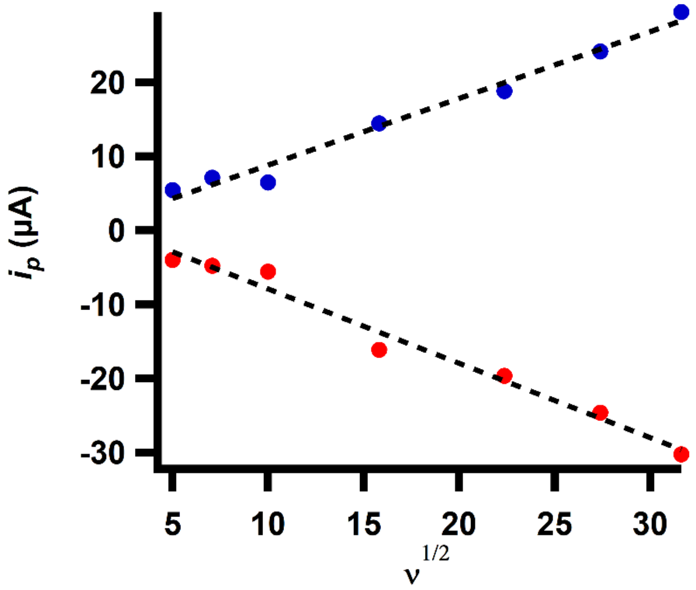

Figure S2. Plot of anodic (blue) and cathodic (red) peak currents (i_p) as a function of the square root of the scan rate for a solution of $Fc^{+/0}$ and Fc* in CH_3CN (0.1 M $TBAPF_6$).

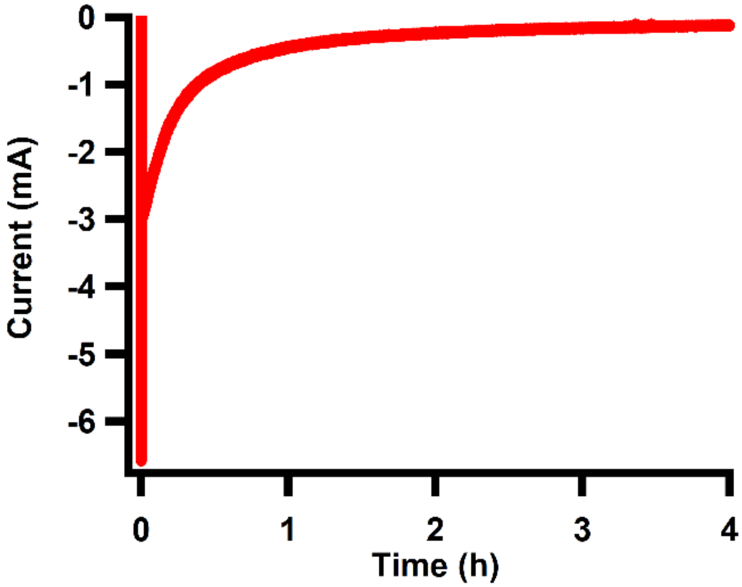

Figure S3. Representative current over time trace during electrolysis. Conditions: 1 mM $[Pt(dmpe)_2](PF_6)_2$ (**1**), 40 mM phenol, 40 mM IPr·CO_2, 0.1 M $TBAPF_6$ in CH_3CN.

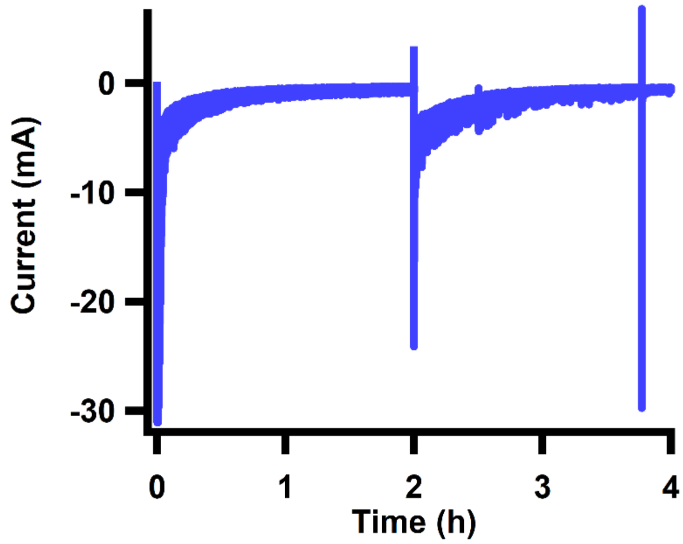

Figure S4. Current over time trace of an electrolysis experiment. The first two hours represent the current passed for 1 mM $[Pt(dmpe)_2](PF_6)_2$ (**1**), 40 mM IPr·CO_2, 40 mM phenol, 0.1 M $TBAPF_6$ in CH_3CN. After two hours, an additional 40 mM of IPr·CO_2 and 40 mM phenol were added to the solution. The increase in current at 3.8 hours is due to readjusting the cell.

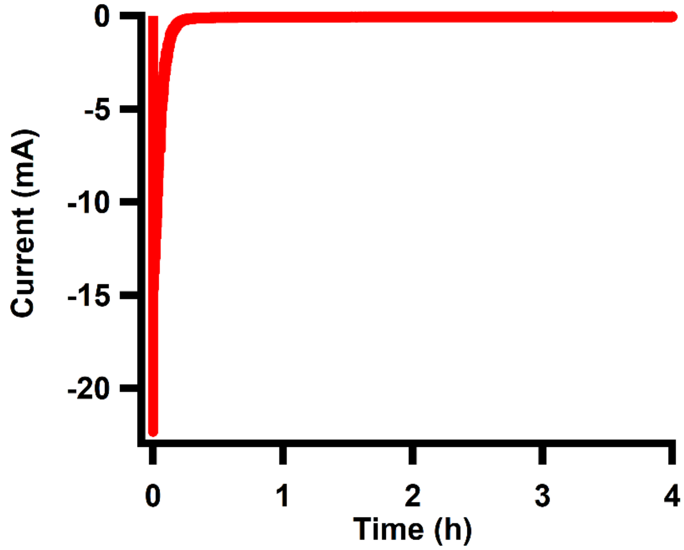

Figure S5. Current over time trace of an electrolysis experiment. Conditions: 1 mM $[Pt(dmpe)_2](PF_6)_2$ (**1**), 40 mM phenol, 40 mM IPr, 0.1 M $TBAPF_6$ in CH_3CN

Determination of k_{obs} for IPr·CO_2 Binding to $[Pt(dmpe)_2](PF_6)_2$. The k_{obs} for IPr·CO_2 binding was found by a series of varying scan rate CVs. 1 mM $[Pt(dmpe)_2](PF_6)_2$ (**1**), 20 mM IPr·CO_2 , and 0.1 M $TBAPF_6$ were dissolved in CH_3CN. The scan rate was increased until the CV scan was reversible (Figure S6). The ratio of the anodic peak and cathodic peak currents was plotted against the natural logarithm of the scan rate yielding a sigmoidal plot. The sigmoidal plot was fitted with a 3rd order polynomial. The half-life was determined by using the scan rate found at the halfway point of the curve and the mV it took to scan from the cathodic to anodic peak. Half-life was used

to find the k_{obs} through the equation below. The experiment was run in triplicate leading to a k_{obs} of 0.40 ± 0.04 s^{-1}.[4–7]

$$t_{\frac{1}{2}} = \frac{\ln(2)}{k_{obs}}$$

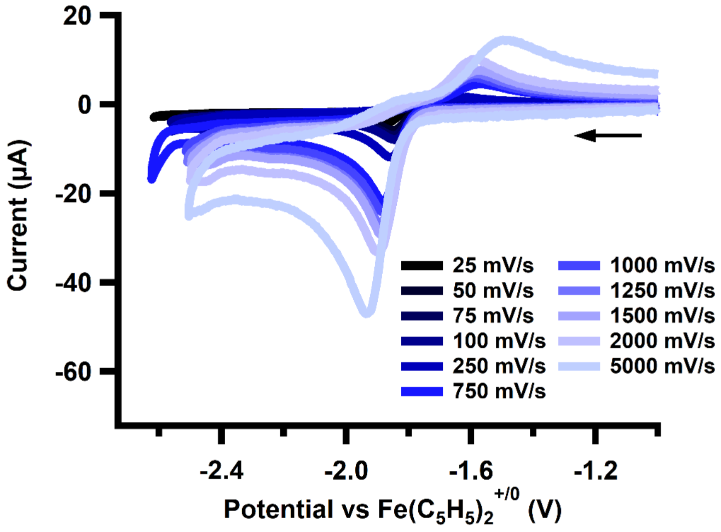

Figure S6. CV data of $[Pt(dmpe)_2](PF_6)_2$ (**1**) (1 mM) and IPr·CO_2 (20 mM) at varying scan rates in CH_3CN (0.1 M $TBAPF_6$). The cathodic shift in potential at higher scan rates occurs in CVs with $[Pt(dmpe)_2](PF_6)_2$ (**1**) due to slow electron transfer.[8]

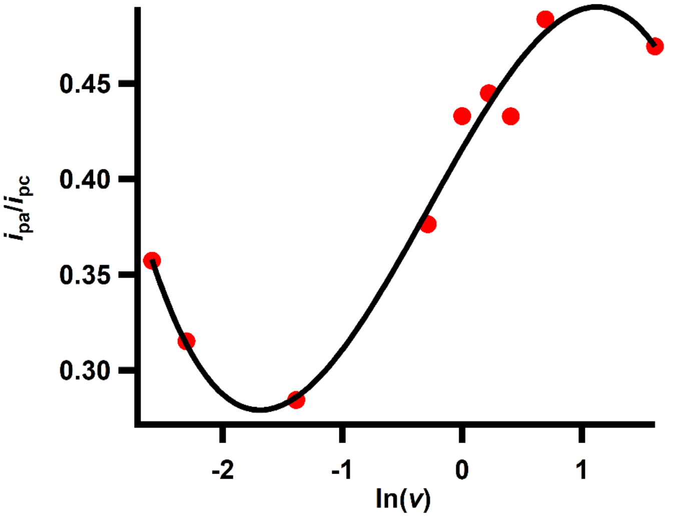

Figure S7. Determination of k_{obs} (1 mM $[Pt(dmpe)_2](PF_6)_2$ (**1**), 20 mM IPr·CO_2 , 0.1 mM $TBAPF_6$ in CH_3CN). The half-life was determined to be 1.67 s, leading to a k_{obs} of 0.41 s^{-1}.

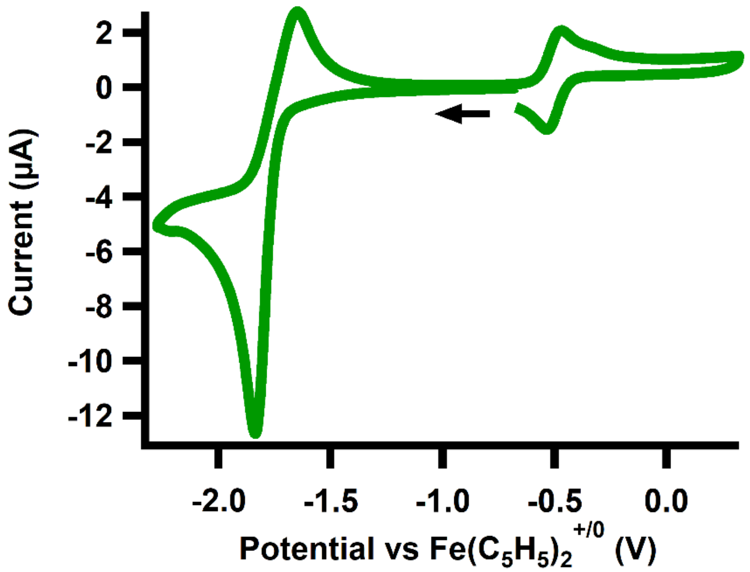

Figure S8. CV of $[Pt(dmpe)_2](PF_6)_2$ (**1**) in CH_3CN (1 mM, 0.1 M $TBAPF_6$, 100 mV/s).

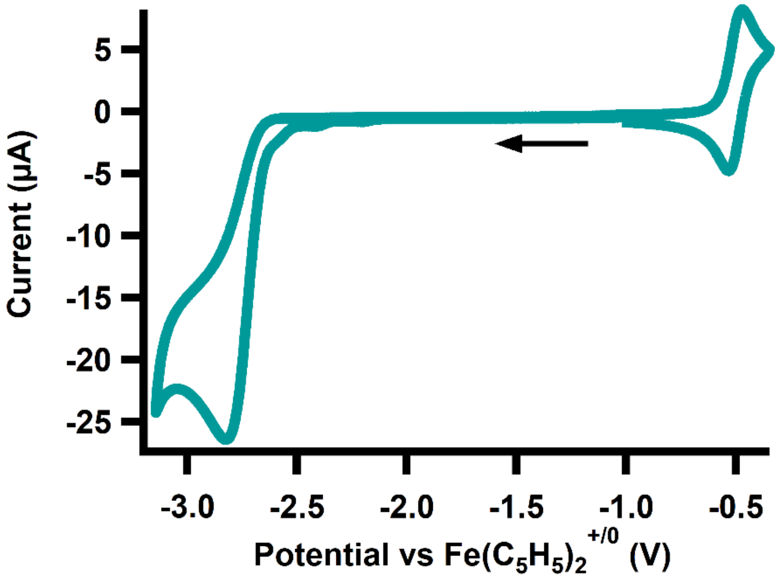

Figure S9. CV of IPr·CO_2 in CH_3CN (1 mM, 0.1 M $TBAPF_6$, 100 mV/s).

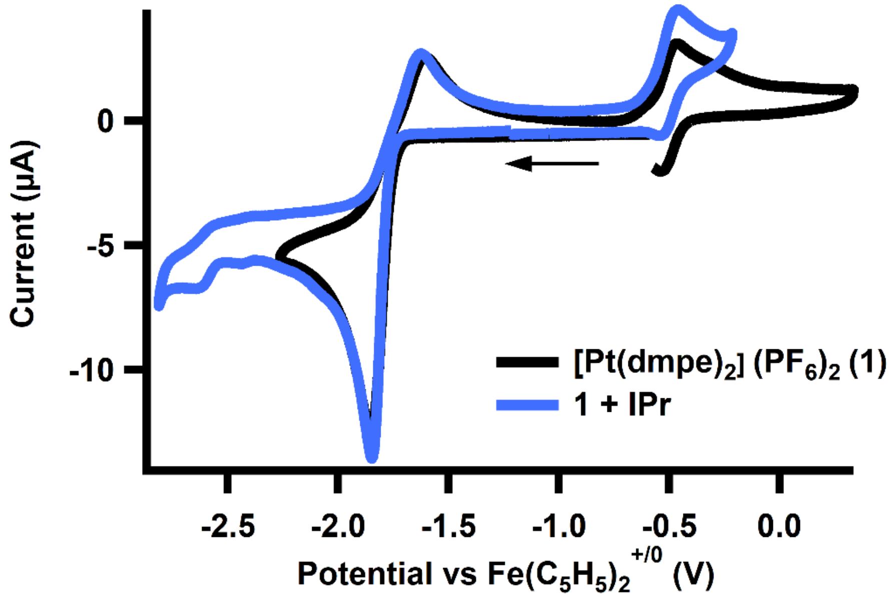

Figure S10. CV of [Pt(dmpe)$_2$](PF$_6$)$_2$ (**1**) (1 mM) and IPr (40 mM) (0.1 M TBAPF$_6$, CH_3CN, 100 mV/s).

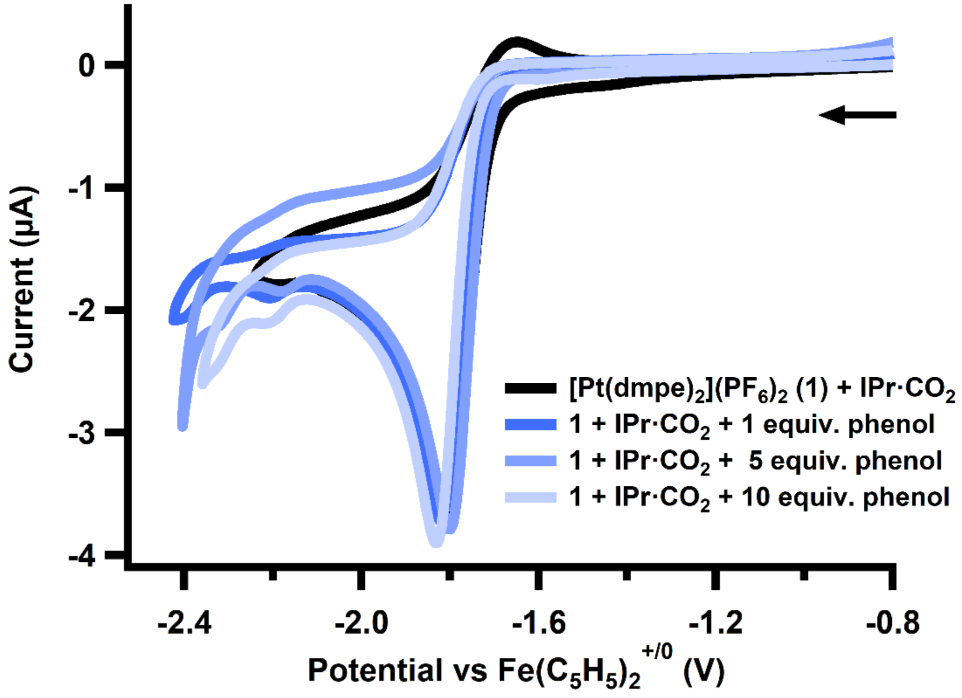

Figure S11. CV of [Pt(dmpe)$_2$](PF$_6$)$_2$ (**1**) (1 mM) and IPr·CO_2 (20 mM) while titrating in phenol (0.1 M TBAPF$_6$, CH_3CN, 100 mV/s).

NMR Spectroscopy

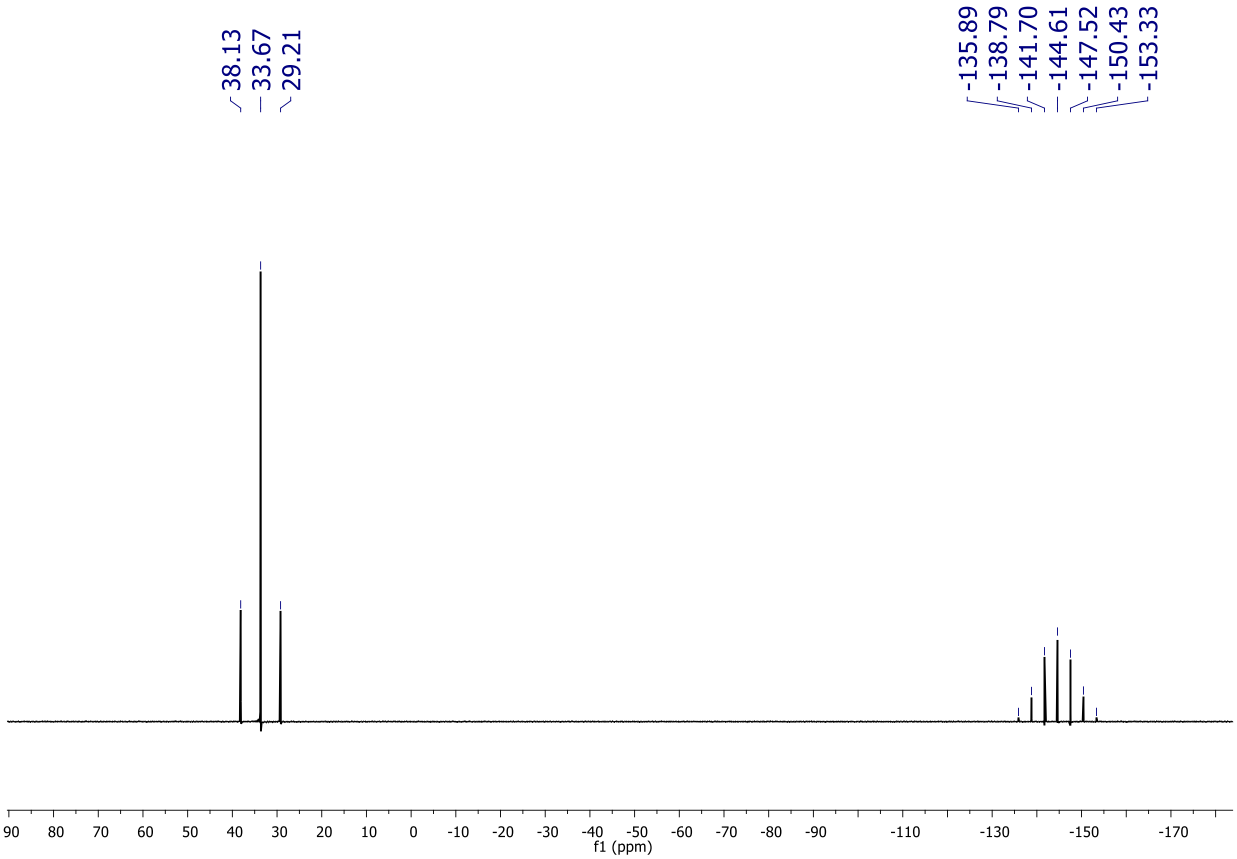

Figure S12. $^{31}P\{^{1}H\}$ NMR spectrum (243 MHz, CD_3CN, 25° C) of $[Pt(dmpe)_2](PF_6)_2$ (**1**). The resonance at 33.7 ppm corresponds to **1** and the septet at -144.6 is the PF_6^- anion.

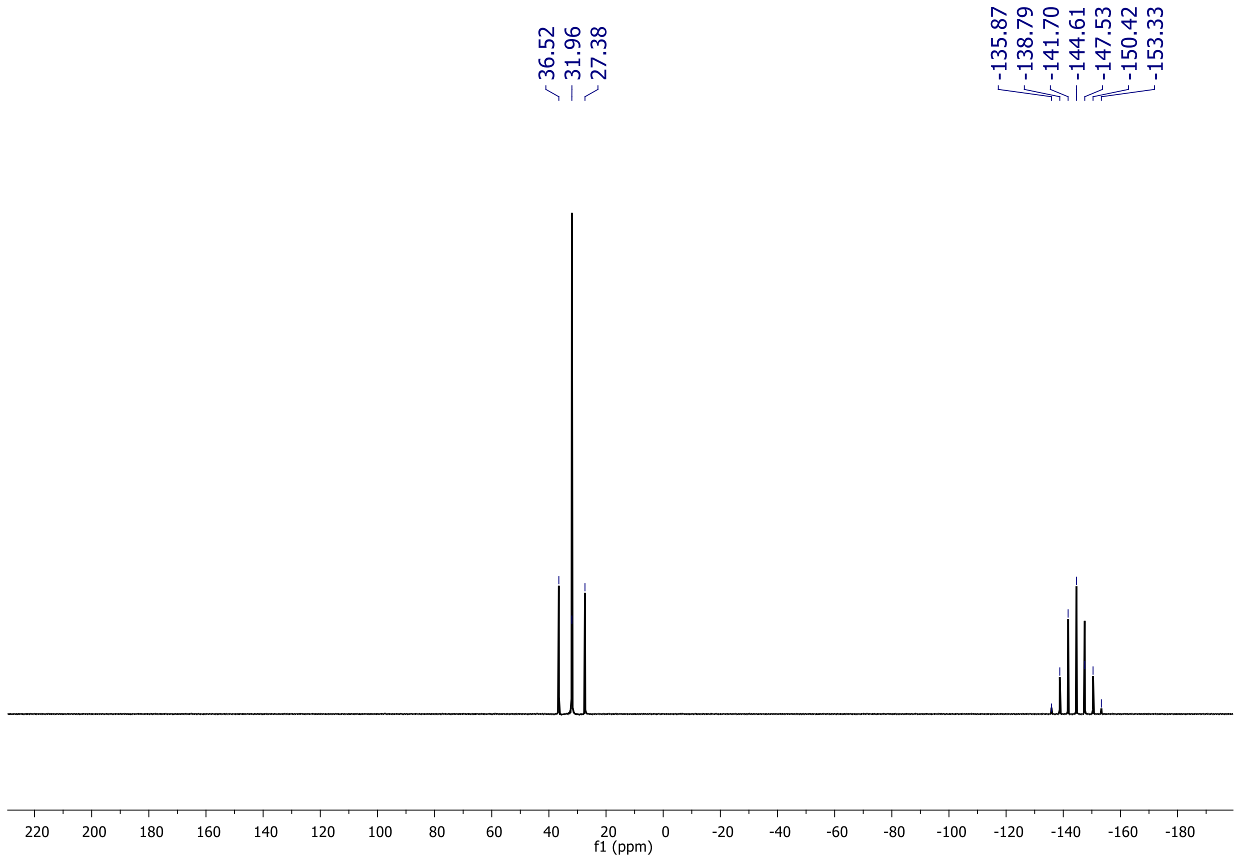

Figure S13. $^{31}P\{^{1}H\}$ NMR spectrum (243 MHz, CD_3CN, 25 °C) of equimolar $[Pt(dmpe)_2](PF_6)_2$ (**1**) and IPr·CO_2. The resonance at 31.96 ppm corresponds to **1** and the septet at -144.6 ppm corresponds to the PF_6^- anion. The lack of other ^{31}P resonances indicates no loss of complex to a paramagnetic species.

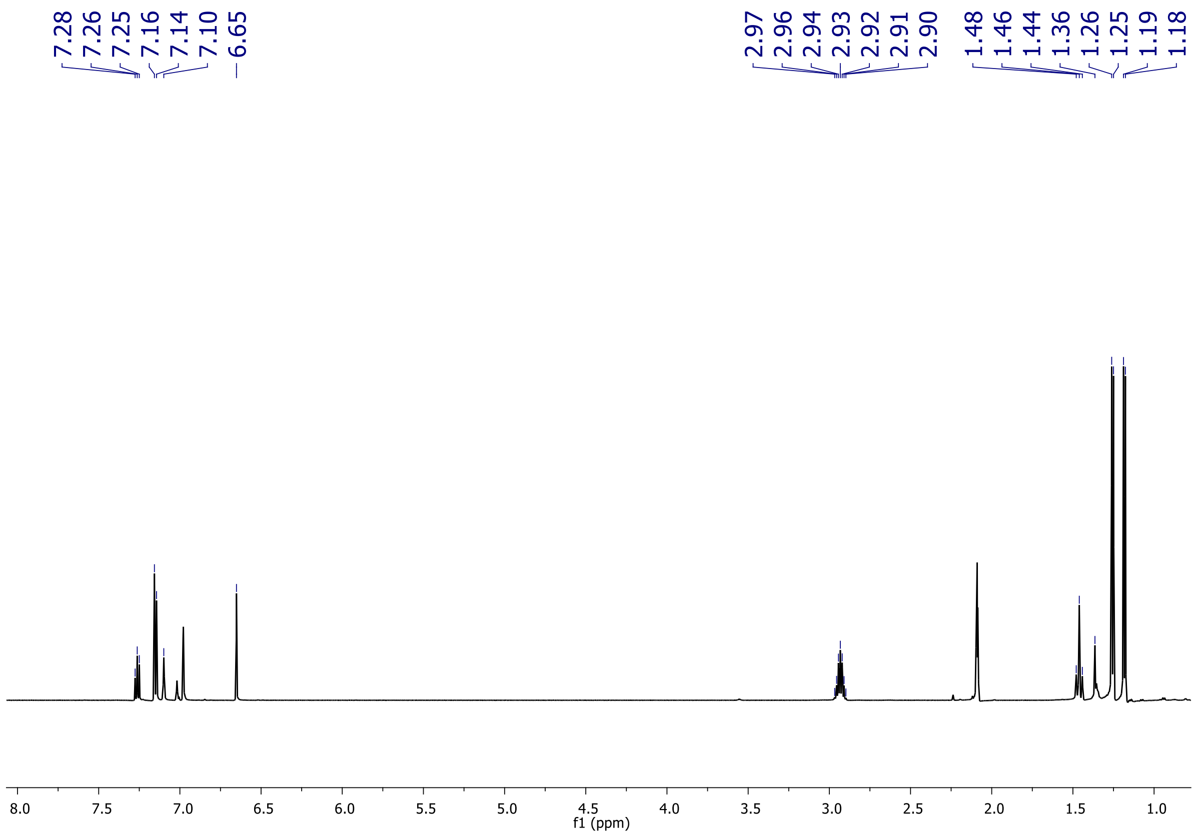

Figure S14. ^{1}H NMR spectrum (600 MHz, CD_3CN, 25 °C) of equimolar $[Pt(dmpe)_2](PF_6)_2$ (**1**) and IPr·CO_2. IPr·CO_2 δ: 1.18 (d), 1.25 (d), 1.36 (m), 1.46 (t), 2.93 (m), 6.65 (s), 7.10 (s), 7.14 (s), 7.26 (t).

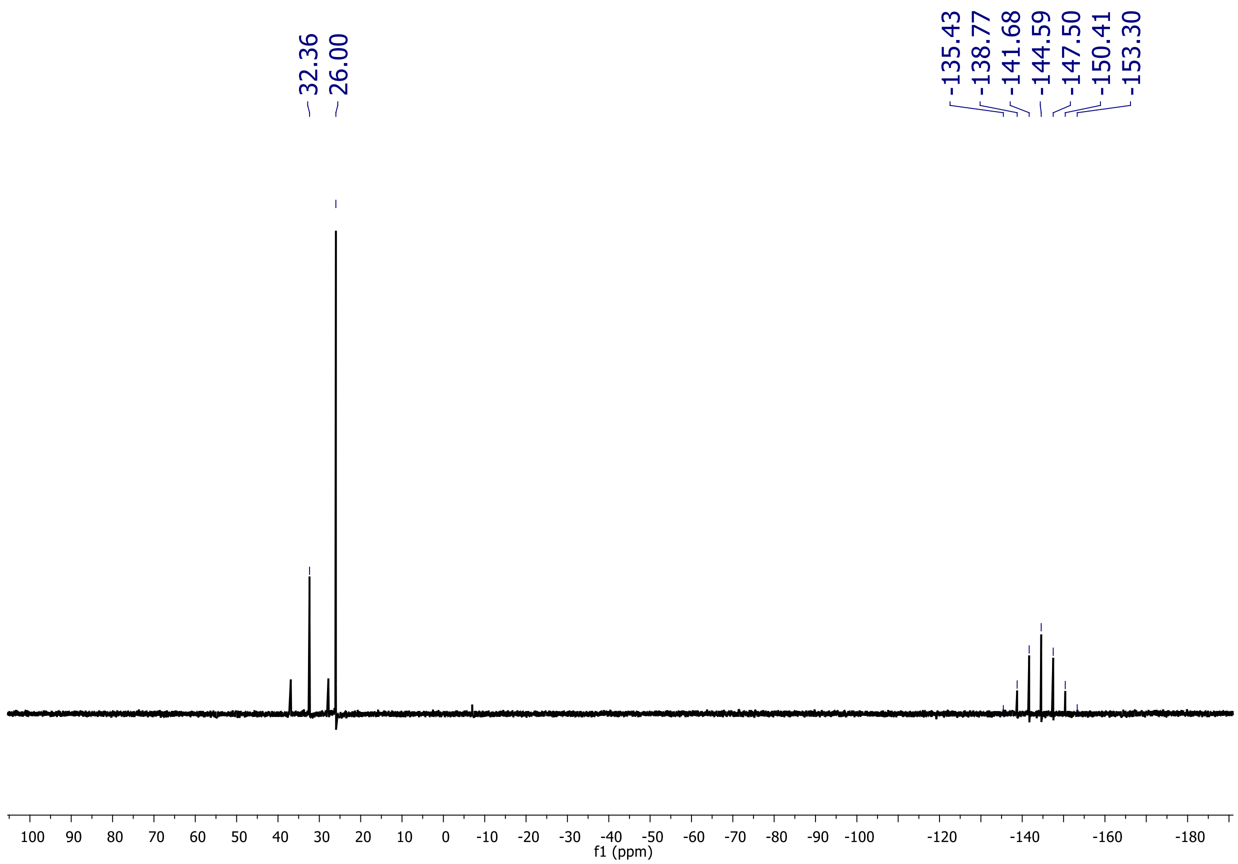

Figure S15. $^{31}P\{^{1}H\}$ NMR spectrum (243 MHz, CD_3CN, 25 °C) of equimolar $[Pt(dmpe)_2](PF_6)_2$ (**1**) and IPr with a triphenylphosphine oxide standard at 26.0 ppm. $[Pt(dmpe)_2](PF_6)_2$ (**1**) is seen at 32.36 ppm and the PF_6^- anion is a septet at -144.6 ppm. The lack of other ^{31}P NMR resonances and the use of an internal standard indicate no loss of complex to a paramagnetic species.

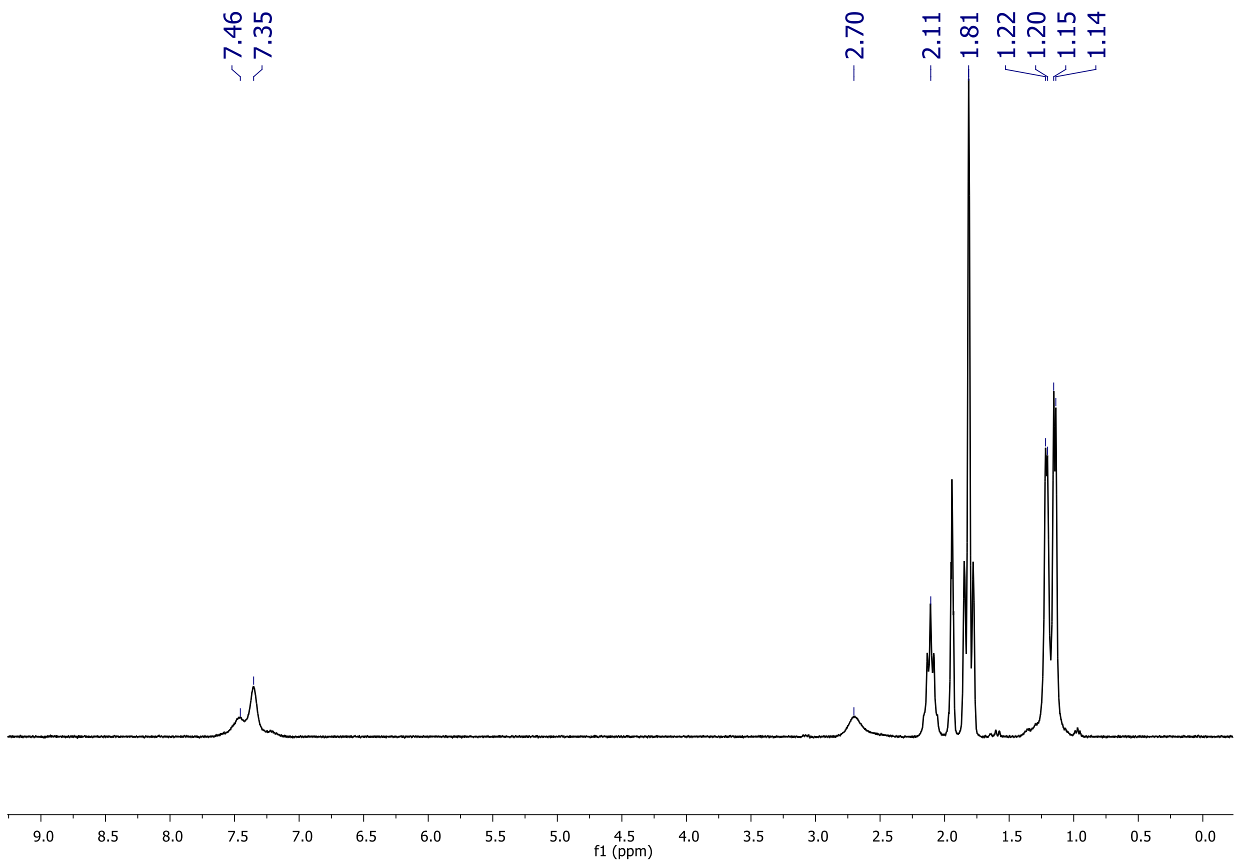

Figure S16. ^{1}H NMR spectrum (400 MHz, CD_3CN, 25 °C) of equimolar $[Pt(dmpe)_2](PF_6)_2$ (**1**) and IPr. IPr δ: 1.14 (d), 1.20 (d), 2.70 (s), 7.35 (s), 7.46 (s). $[Pt(dmpe)_2](PF_6)_2$ (**1**) δ: 2.11 (t)

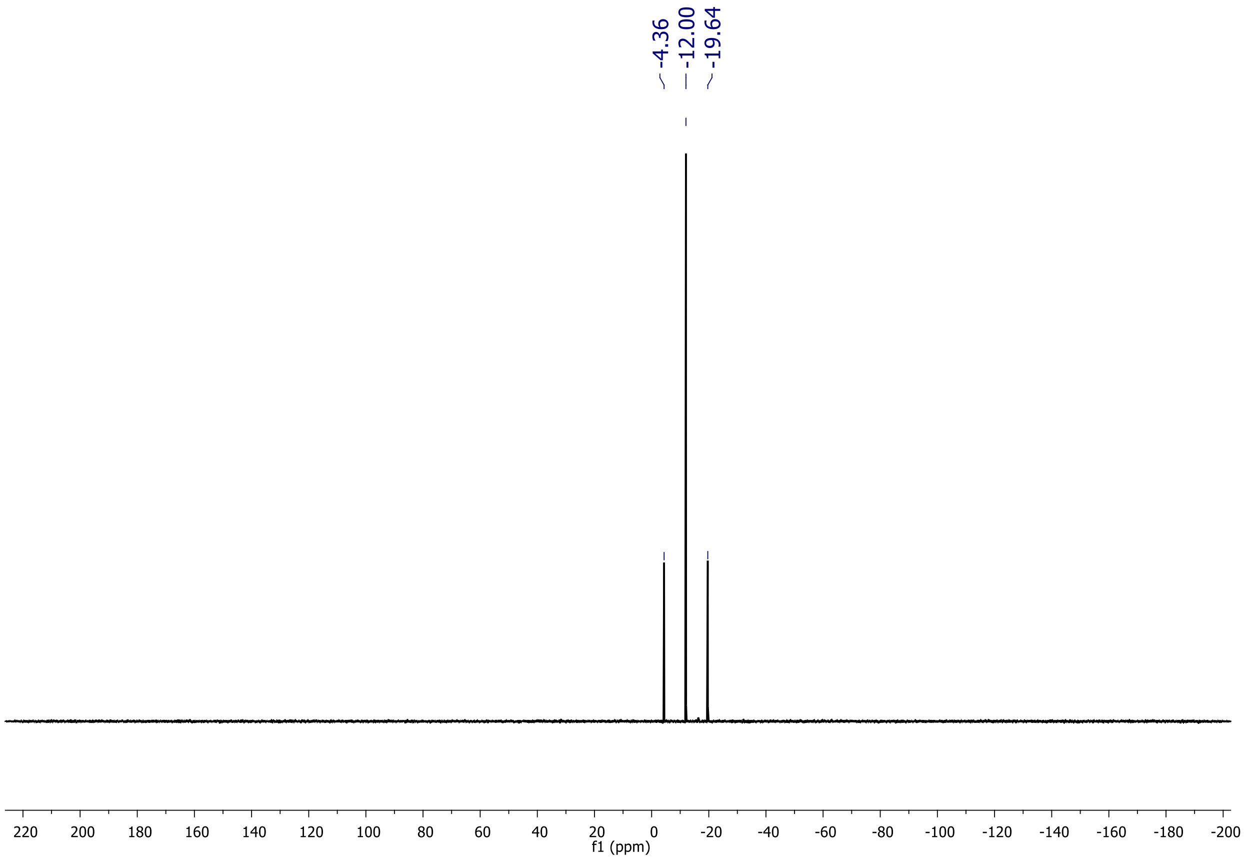

Figure S17. $^{31}P\{^{1}H\}$ NMR spectrum (243 MHz, toluene-d_8, 25 °C) of Pt(dmpe)$_2$ (**2**) showing a resonance at -12.0 ppm.

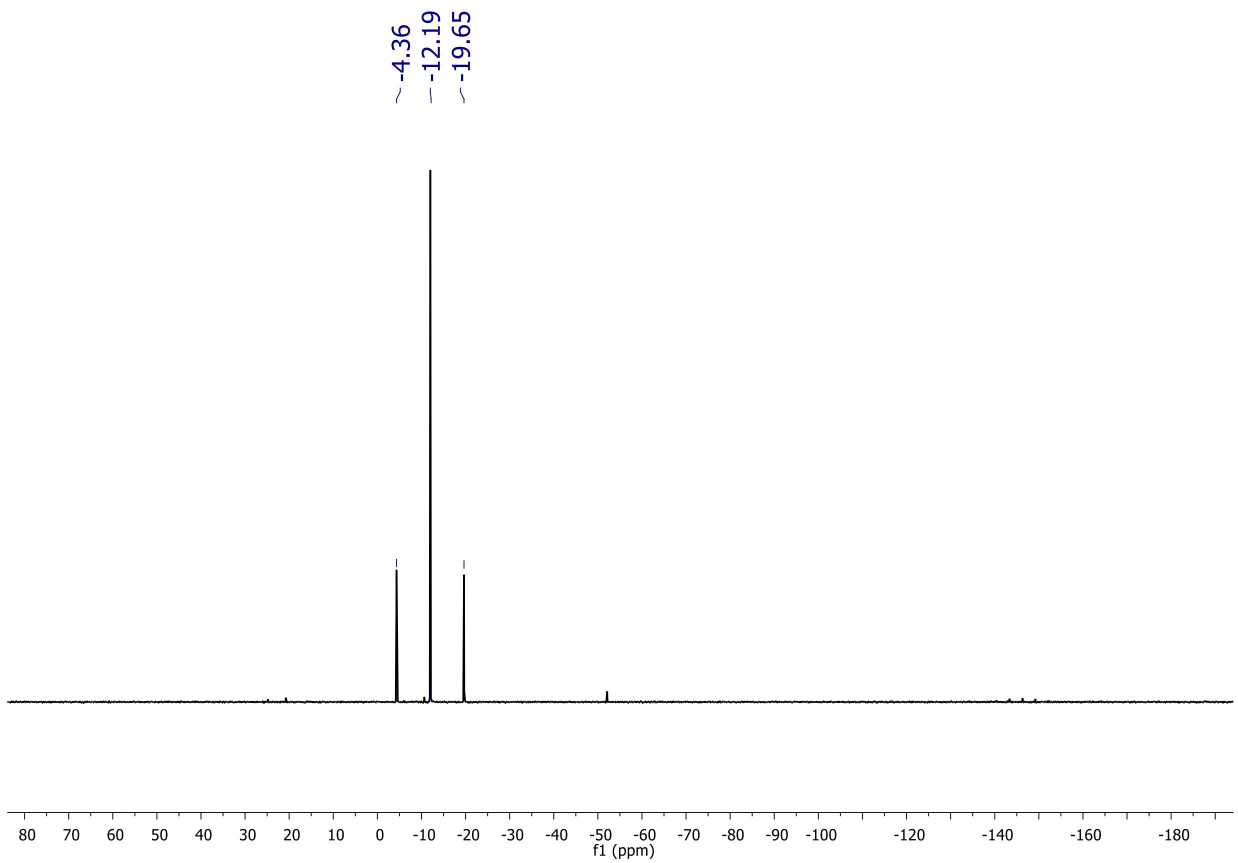

Figure S18. $^{31}P\{^1H\}$ NMR spectrum (243 MHz, CD_3CN, 25 °C) of equimolar $Pt(dmpe)_2$ (**2**) (-12.2 ppm) and IPr.

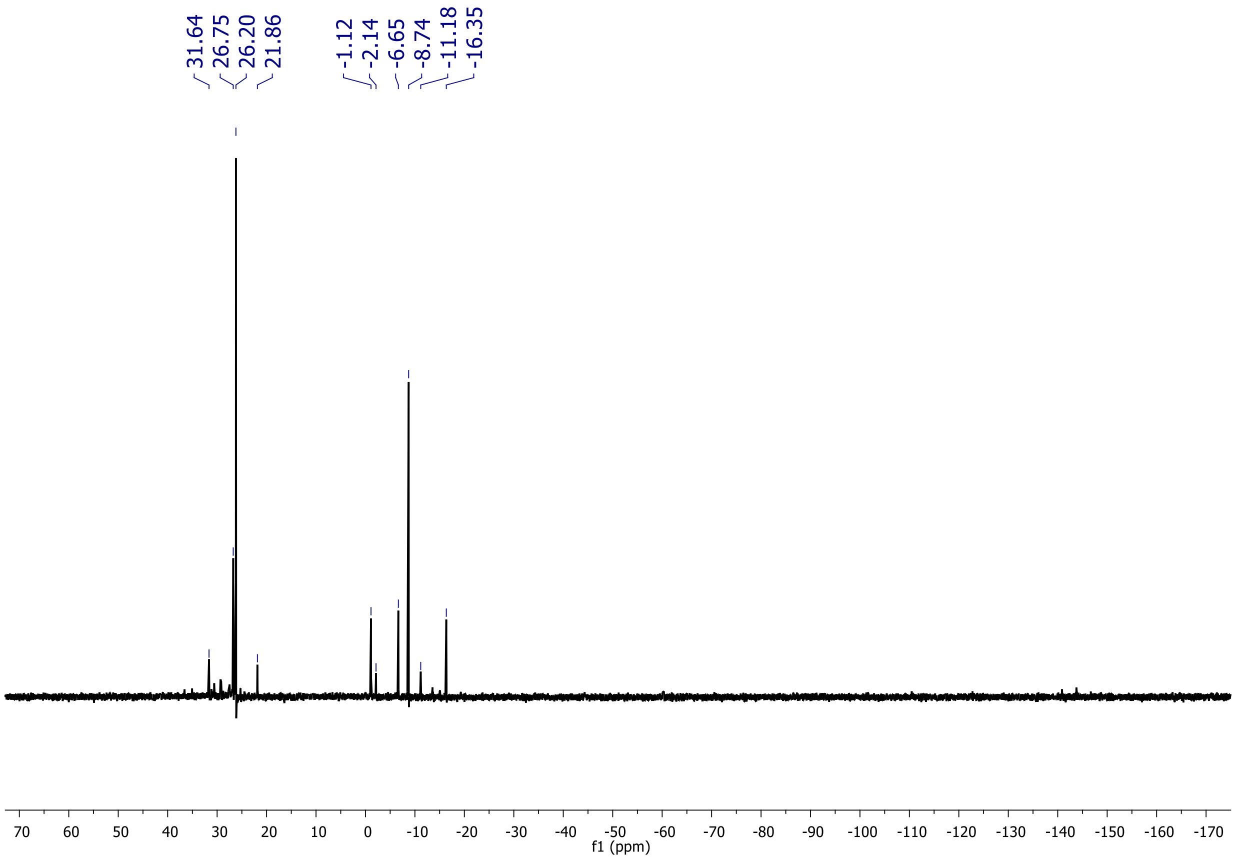

Figure S19. $^{31}P\{^{1}H\}$ NMR spectrum (243 MHz, CD_3CN, toluene-d_8, 25 °C) of equimolar $Pt(dmpe)_2$ (**2**) and IPr·CO_2. $Pt(dmpe)_2$ (**2**) is seen at -8.74 ppm, $[Pt(dmpe)_2](PF_6)_2$ (**1**) impurity is at -6.65 ppm, triphenylphosphine oxide is a singlet at 26.20 ppm, and a new species is observed at 26.75 ppm. Based on the integrations, the new species is equimolar to unreacted $Pt(dmpe)_2$ (**2**).

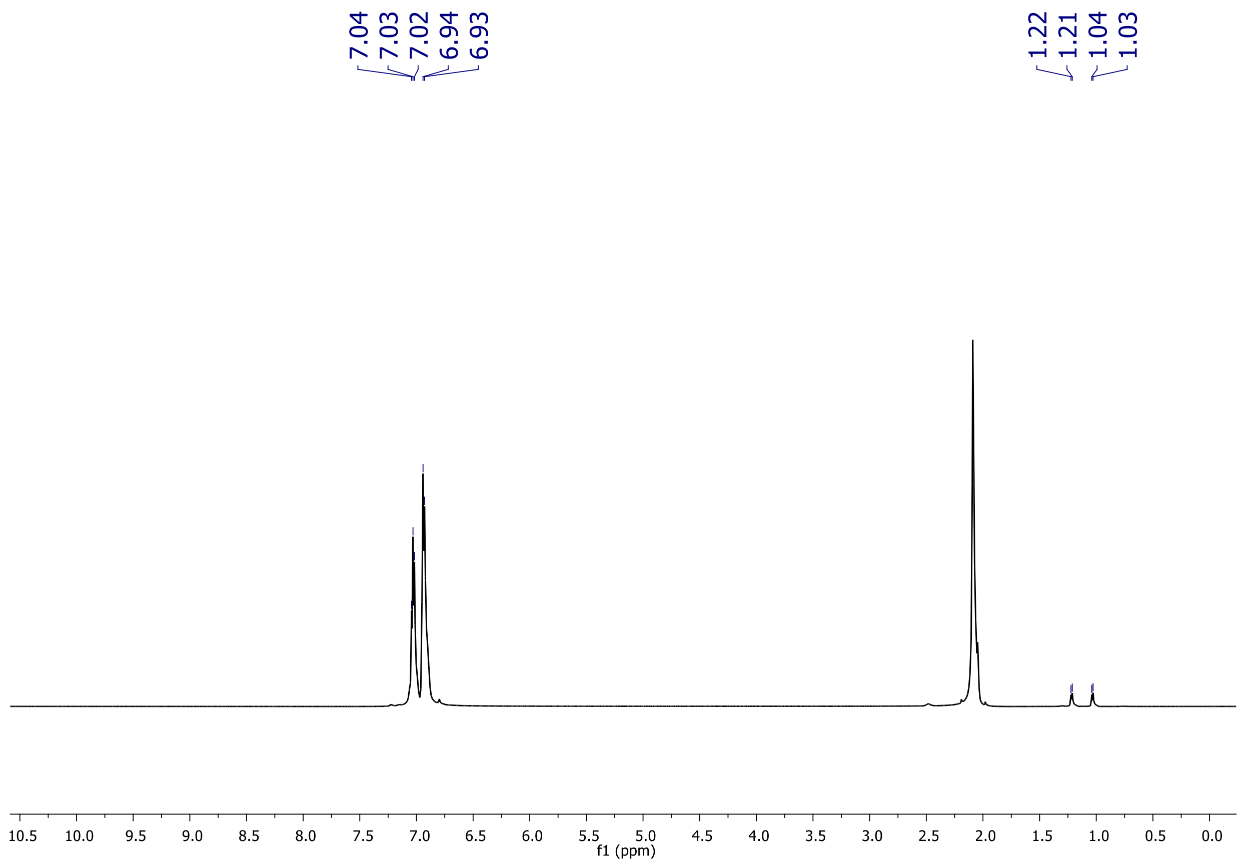

Figure S20. ^{1}H NMR spectrum (400 MHz, CD_3CN, 25 °C) of equimolar Pt(dmpe)$_2$ (**2**) and IPr·CO_2. IPr·CO_2 δ:1.03 (d), 1.21 (d). The aromatic protons for IPr·CO_2 may be under the toluene peaks at 6.93 – 7.04 ppm.

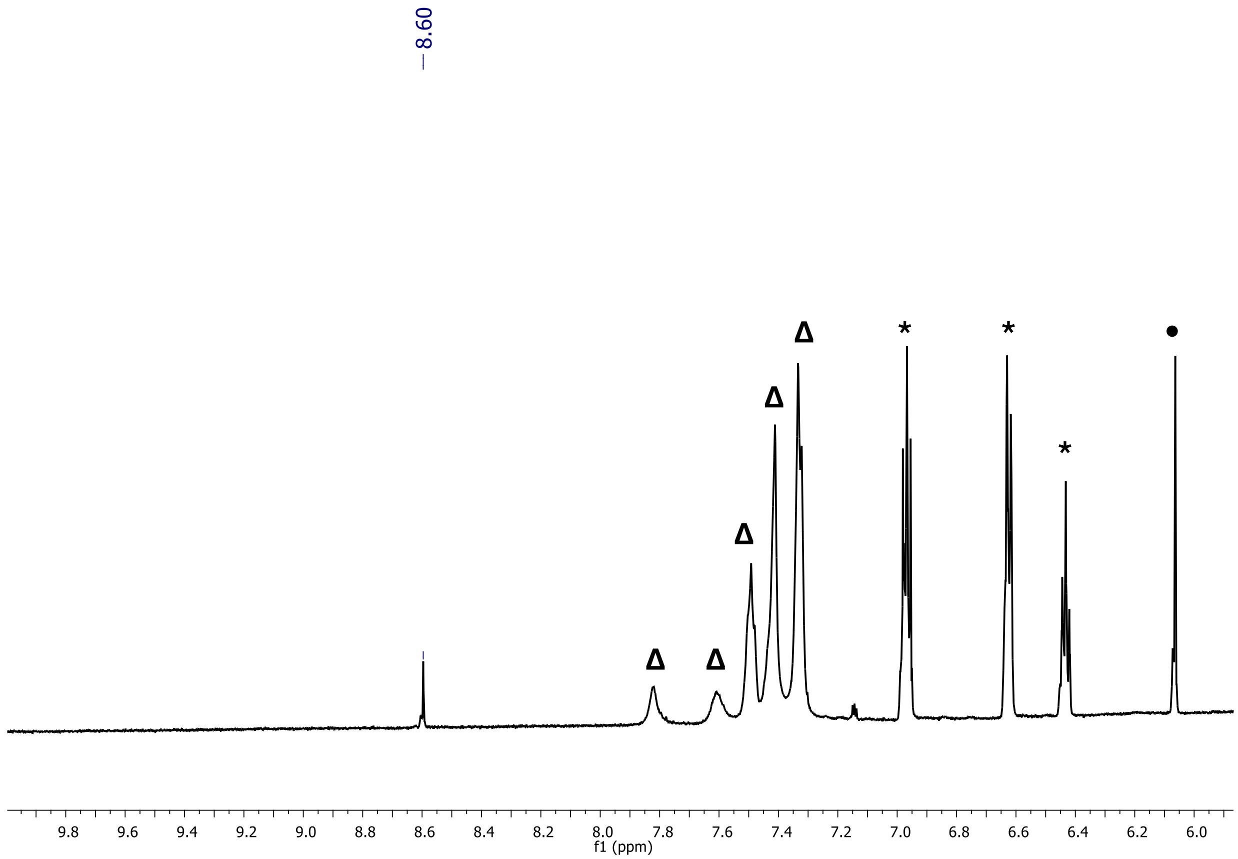

Figure S21. ^{1}H NMR spectrum (600 MHz, CD_3CN, 25 °C) of post-electrolysis solution. Formate corresponds to the singlet at 8.60 ppm. Other resonances shown correspond to IPr·CO_2 (**Δ**), phenol (*), and trimethoxybenzene (•).

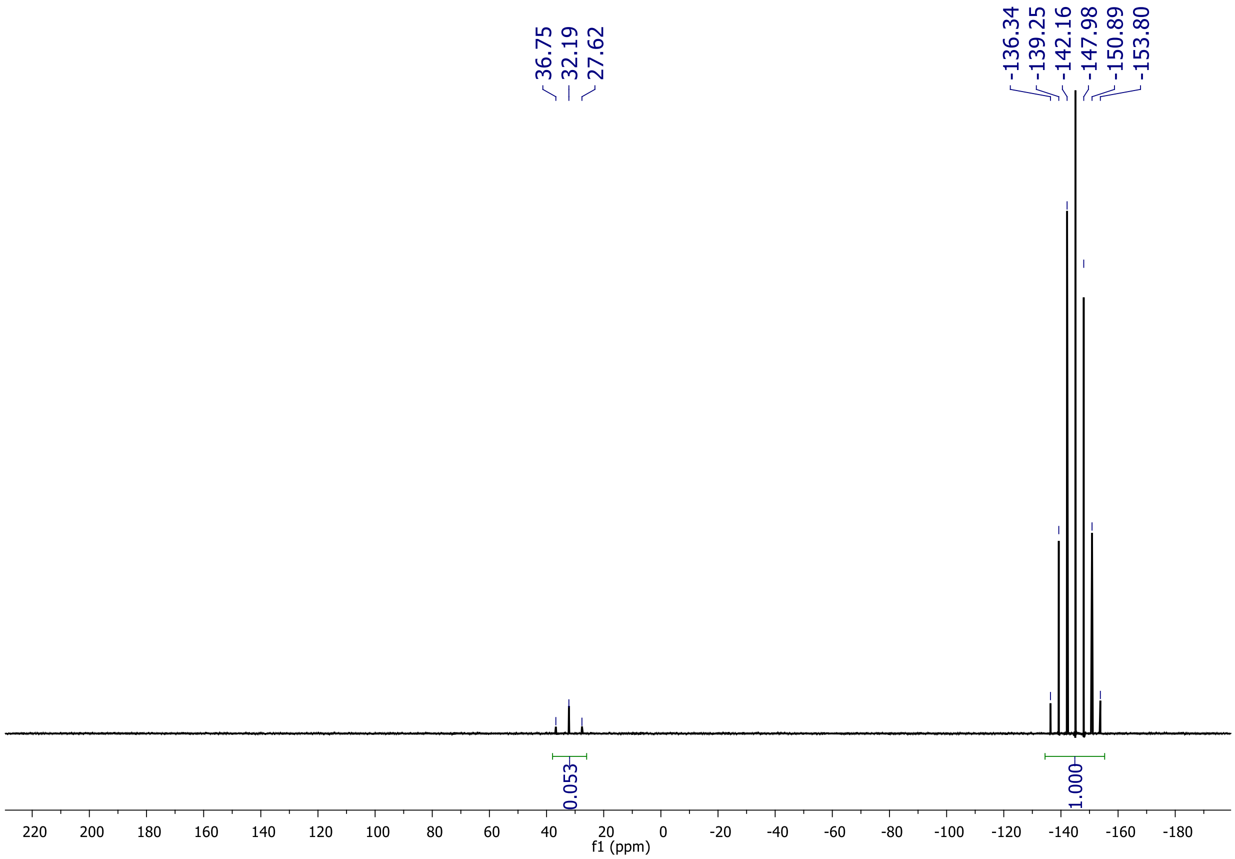

Figure S22. $^{31}P\{^{1}H\}$ NMR spectrum (243 MHz, CD_3CN, 25 °C) of post-electrolysis solution. $[Pt(dmpe)_2](PF_6)_2$ (**1**) is the resonance at 32.2 ppm and the PF_6^- anion is the septet at -145 ppm. Integrations match that of a standard solution of 1 mM $[Pt(dmpe)_2](PF_6)_2$ (**1**) and 100 mM $TBAPF_6$ which indicates no loss of complex to a paramagnetic species.

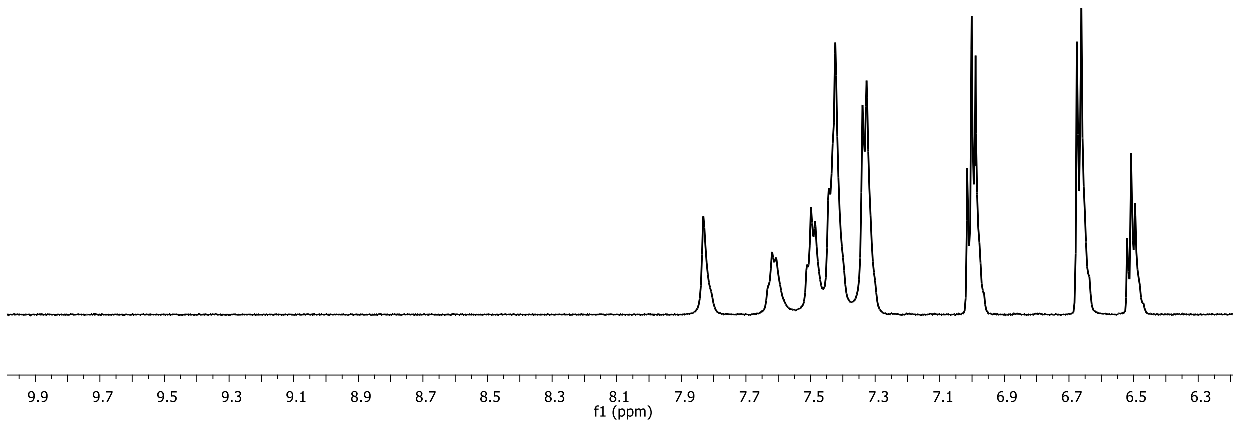

Figure S23. ^{1}H NMR spectrum (600 MHz, CD_3CN, 25 °C) of post-electrolysis solution with no catalyst and 40 mM IPr·CO_2, 40 mM phenol, and 0.1 M $TBAPF_6$ in CH_3CN, demonstrating no formate (normally at 8.6 ppm) is formed without catalyst present.

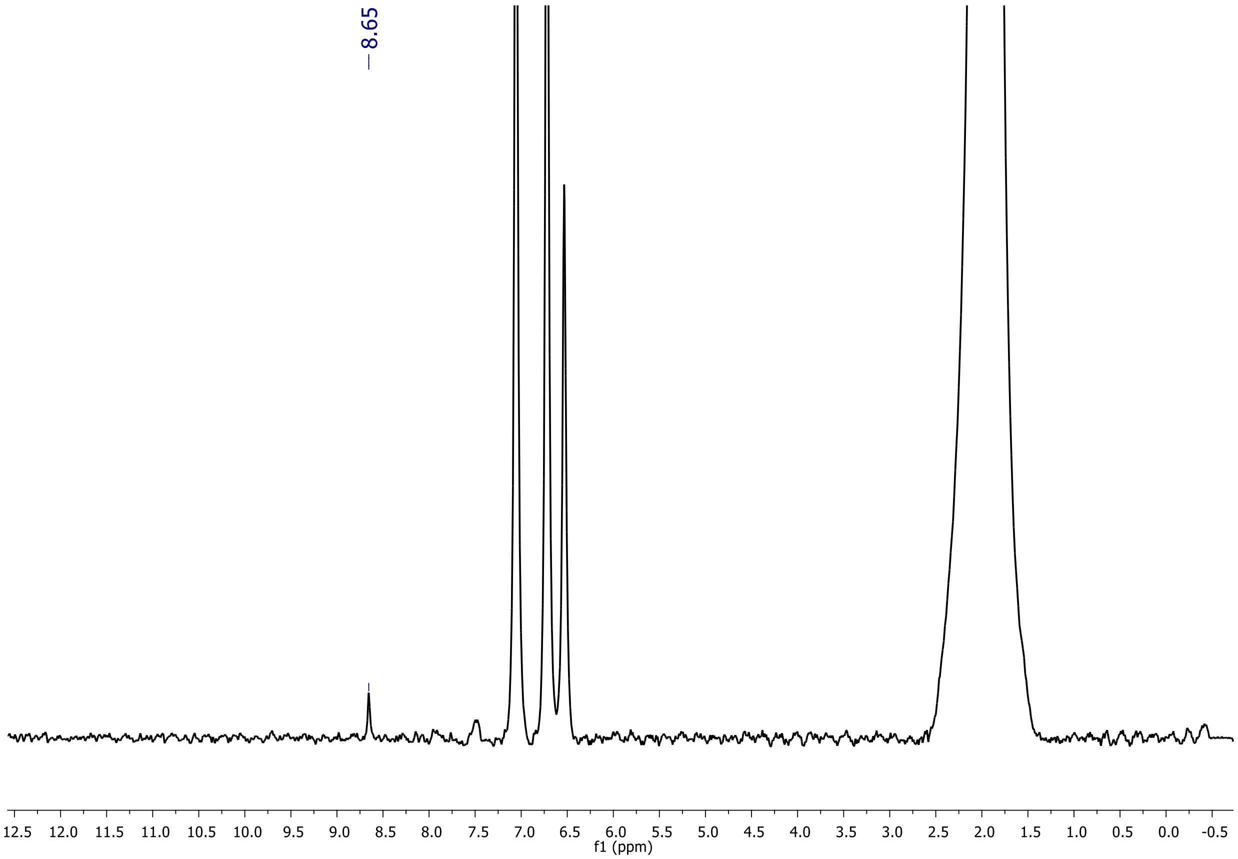

Figure S24. ^{2}H NMR spectrum (92 MHz, CD_3CN, 25 °C) of post-electrolysis solution. The singlet at 8.65 ppm corresponds to DCO_2^-.

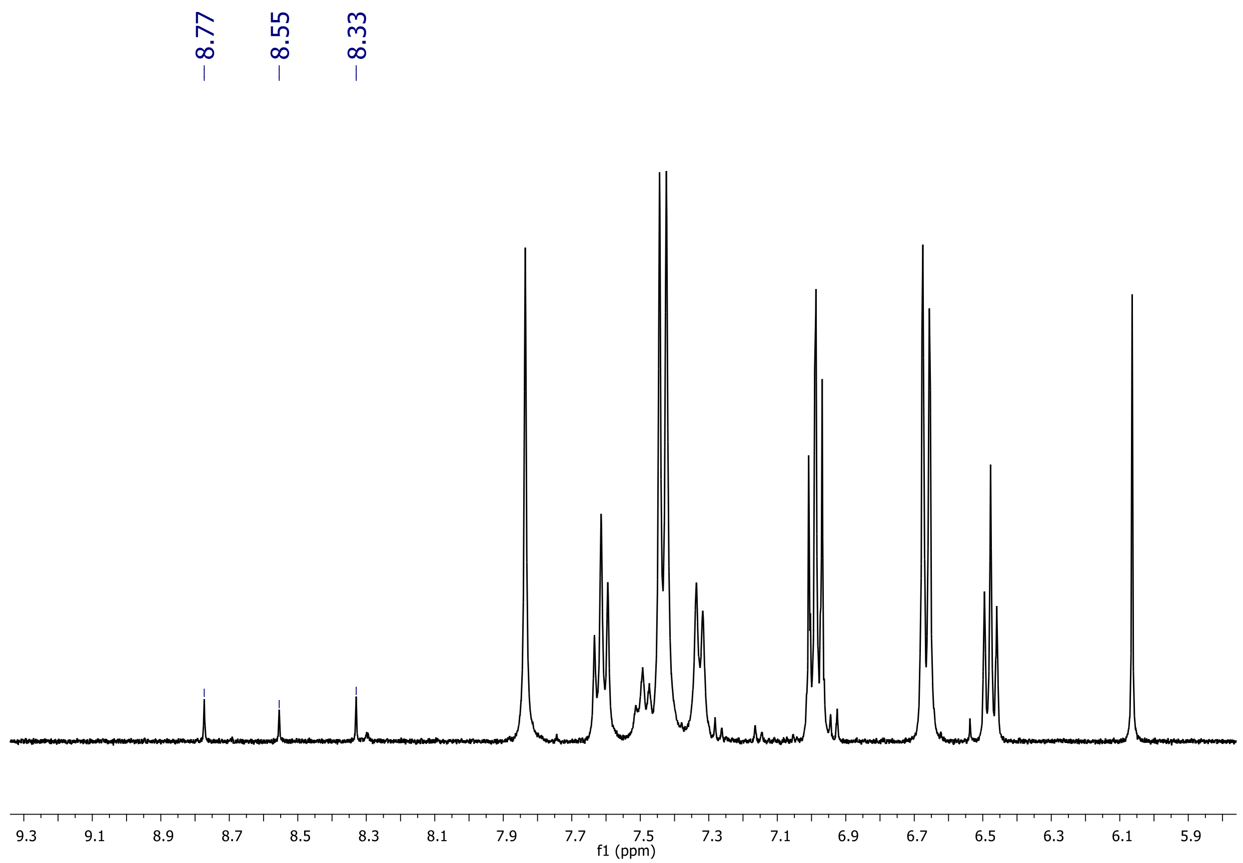

Figure S25. ^{1}H NMR spectrum (400 MHz, CD_3CN, 25 °C) of post-electrolysis solution with 1 mM $[Pt(dmpe)_2](PF_6)_2$ (**1**), 30 mM IPr·$^{13}CO_2$ generated from $^{13}CO_2$, 30 mM phenol, 0.1 M $TBAPF_6$ in CH_3CN. ^{13}C-labelled formate is seen at 8.33 and 8.77 ppm.

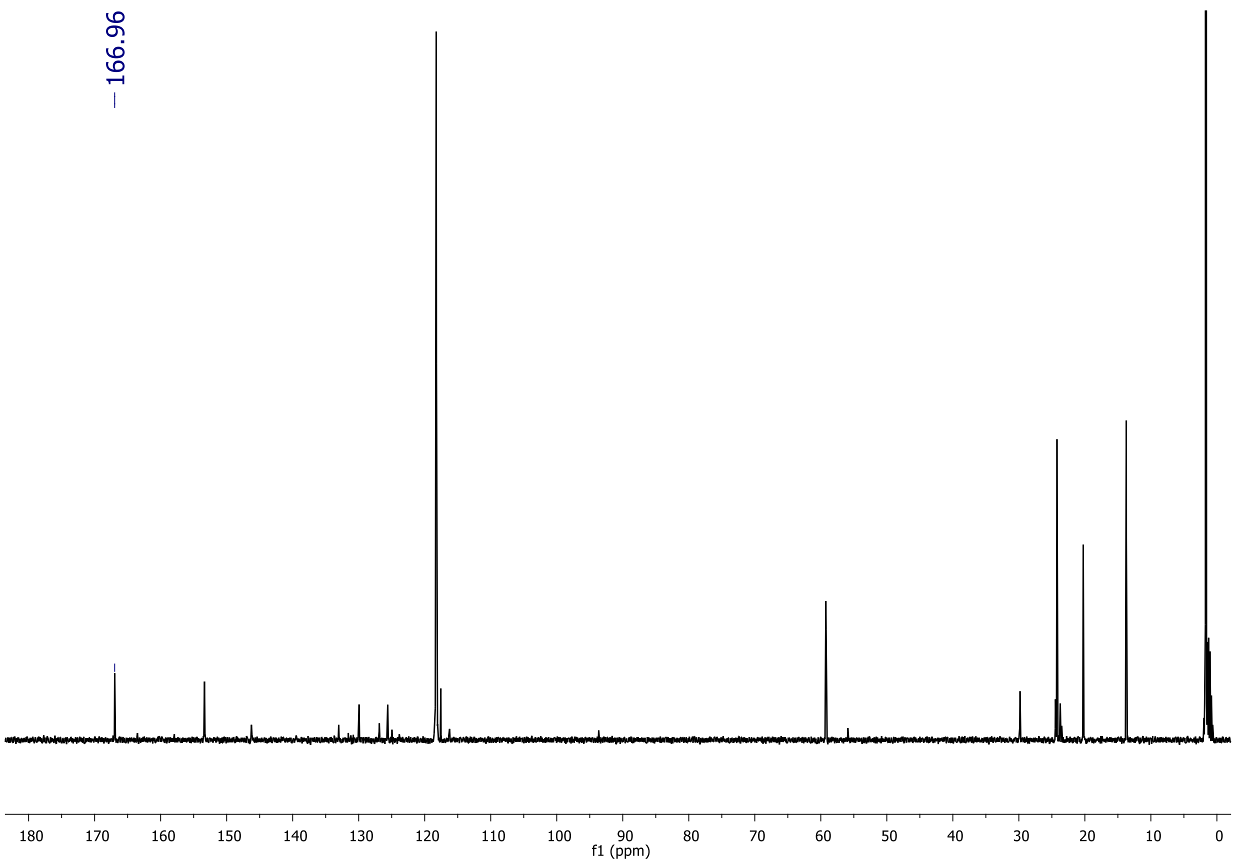

Figure S26. ^{13}C NMR spectrum (92 MHz, CD_3CN, 25 °C) of post-electrolysis solution with 1 mM $[Pt(dmpe)_2](PF_6)_2$ (**1**), 30 mM IPr·$^{13}CO_2$ generated from $^{13}CO_2$, 30 mM phenol, 0.1 M $TBAPF_6$ in CH_3CN. ^{13}C-labeled formate corresponds to the singlet at 167 ppm.

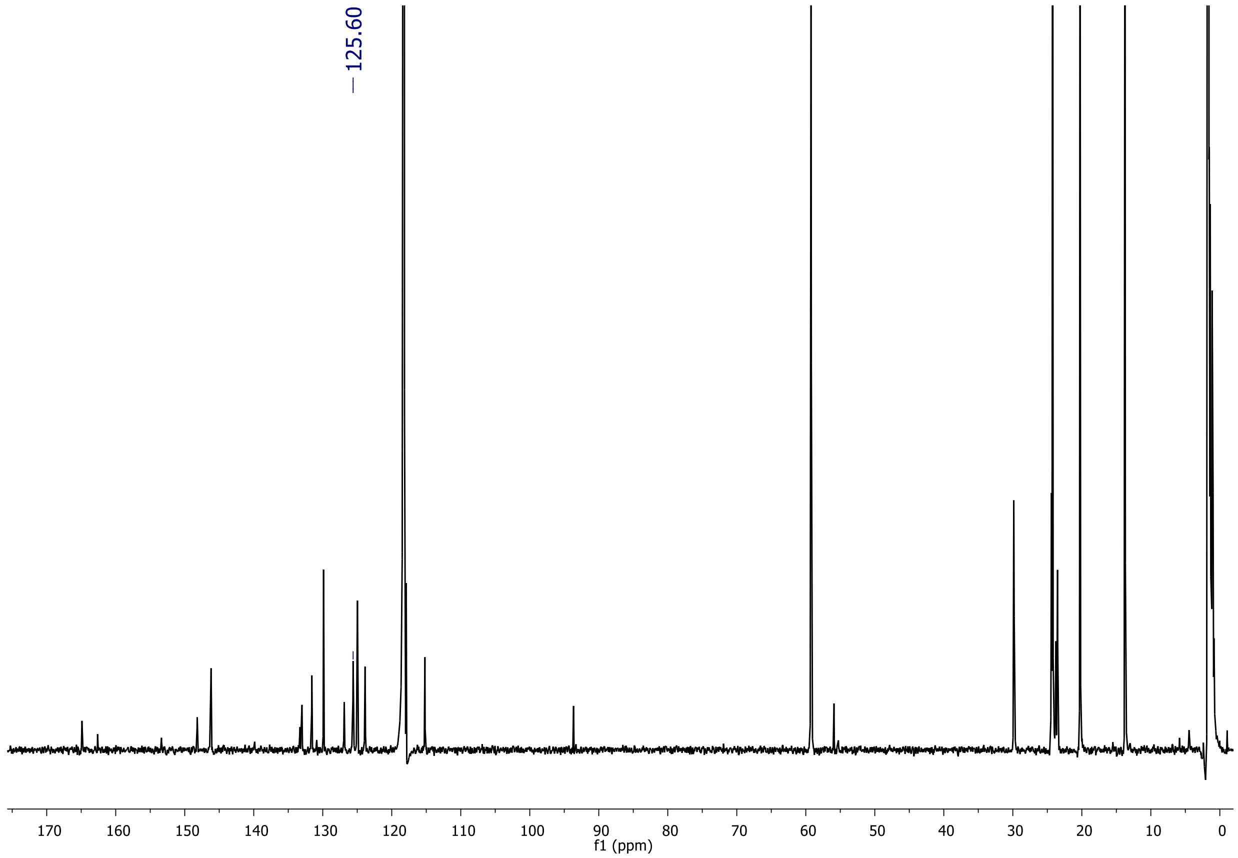

Figure S27. ^{13}C NMR spectrum (151 MHz, CD_3CN, 25 °C) of post-electrolysis solution. CO_2 is seen at 126 ppm.

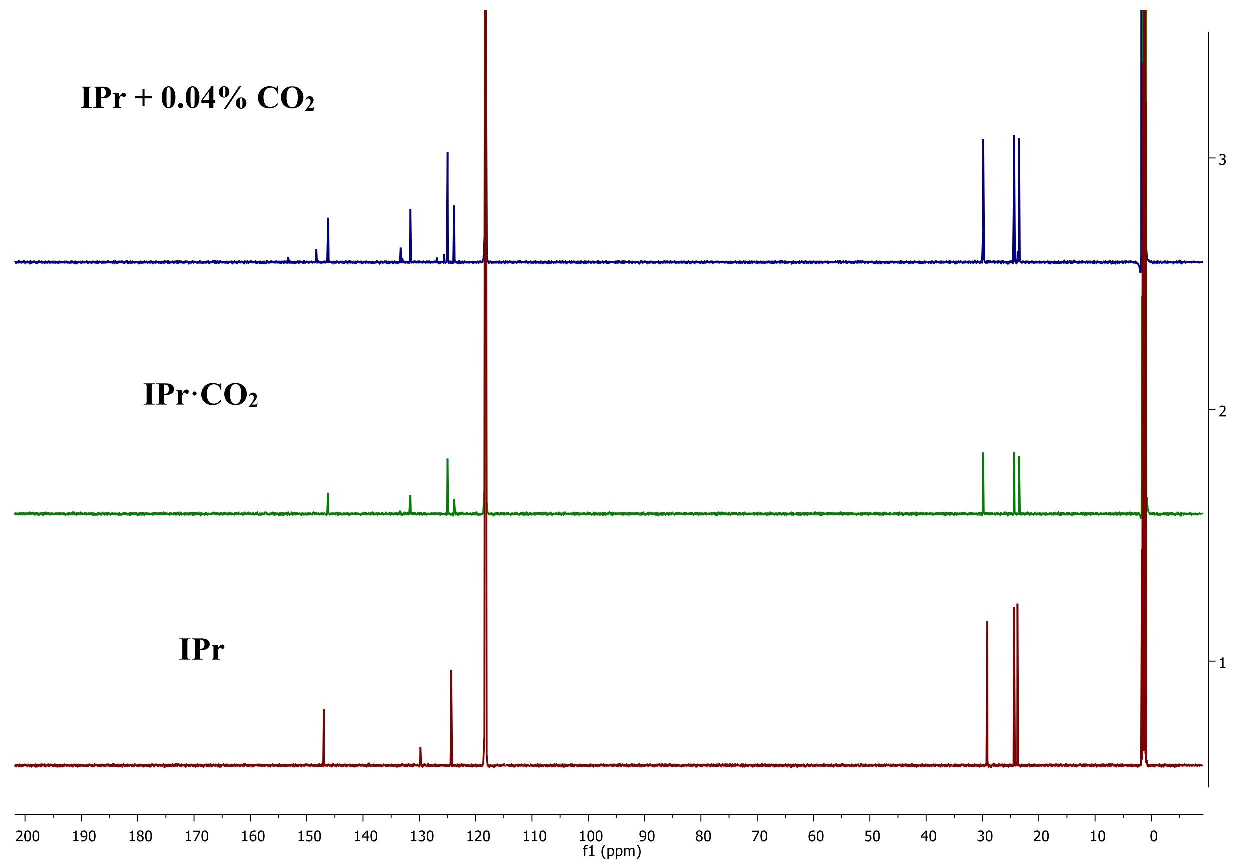

Figure S28. ^{13}C NMR spectra (151 MHz, CD_3CN, 25 °C) of IPr (bottom, red), IPr·CO_2 (middle, green), and IPr + 0.04% CO_2 (top, blue). IPr δ = 23.8, 24.4, 29.1, 124.3, 129.8, 147.0 ppm. IPr·CO_2 δ = 23.5, 24.4, 29.9, 123.8, 125.0, 131.6, 146.2 ppm. IPr + 0.04% CO_2 δ = 23.5, 24.4, 29.9, 123.4, 125.0, 131.6, 133.3, 146.2, 148.3, 153.3 ppm.

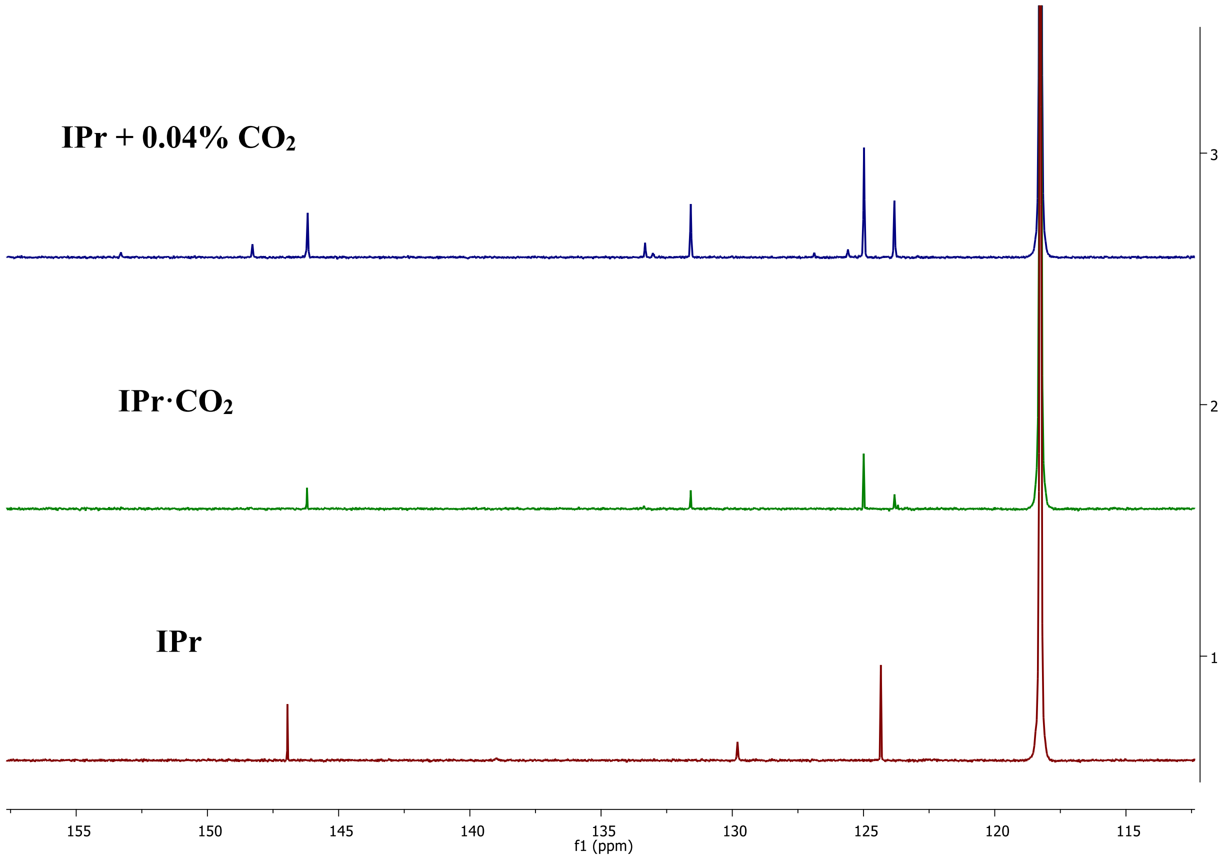

Figure S29. ^{13}C NMR spectra (151 MHz, CD_3CN, 25 °C) inset of Figure S20 (above). Relevant chemical shifts corresponding to the CO_2-bound NHC are 123.4, 125.0, 131.6, 133.3, 146.2, 148.3, 153.3 ppm.

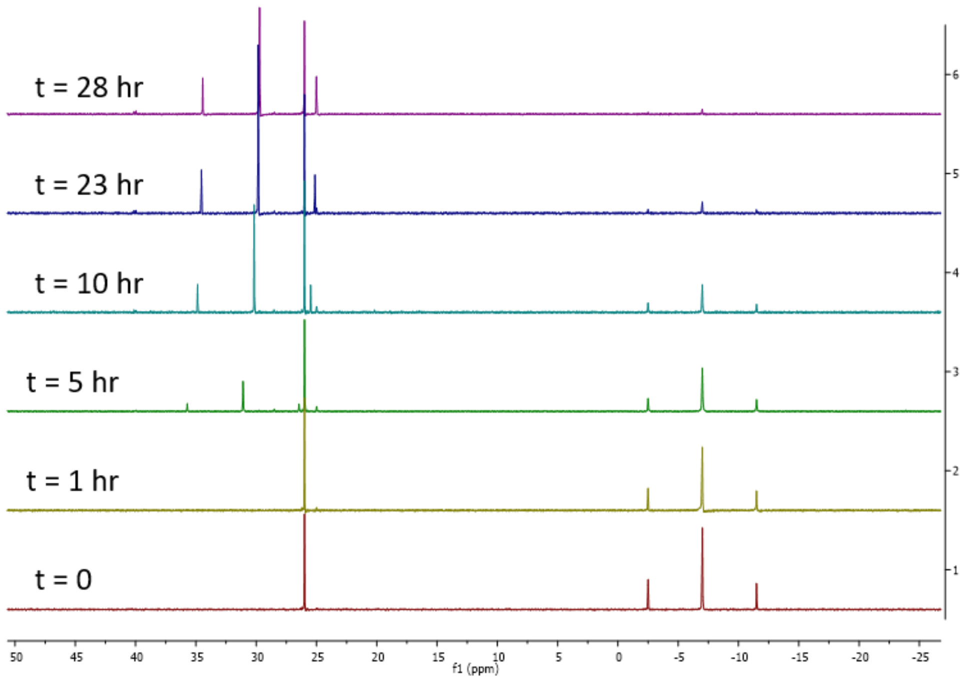

Figure S30. $^{31}P\{^{1}H\}$ NMR spectrum (243 MHz, CD_3CN, 25 °C) of 1 mM $[HPt(dmpe)_2](PF_6)$ (**3**) upon addition of 20 equivalents (20 mM) of $IPr{\cdot}CO_2$ at t = 0, 1, 5, 10, 23, and 28 hours. The resonance at 27 ppm corresponds to the internal standard triphenylphosphine oxide. At t = 28 hours, 94% of the $[HPt(dmpe)_2](PF_6)$ (**3**) has converted to $[Pt(dmpe)_2](PF_6)_2$ (**1**).

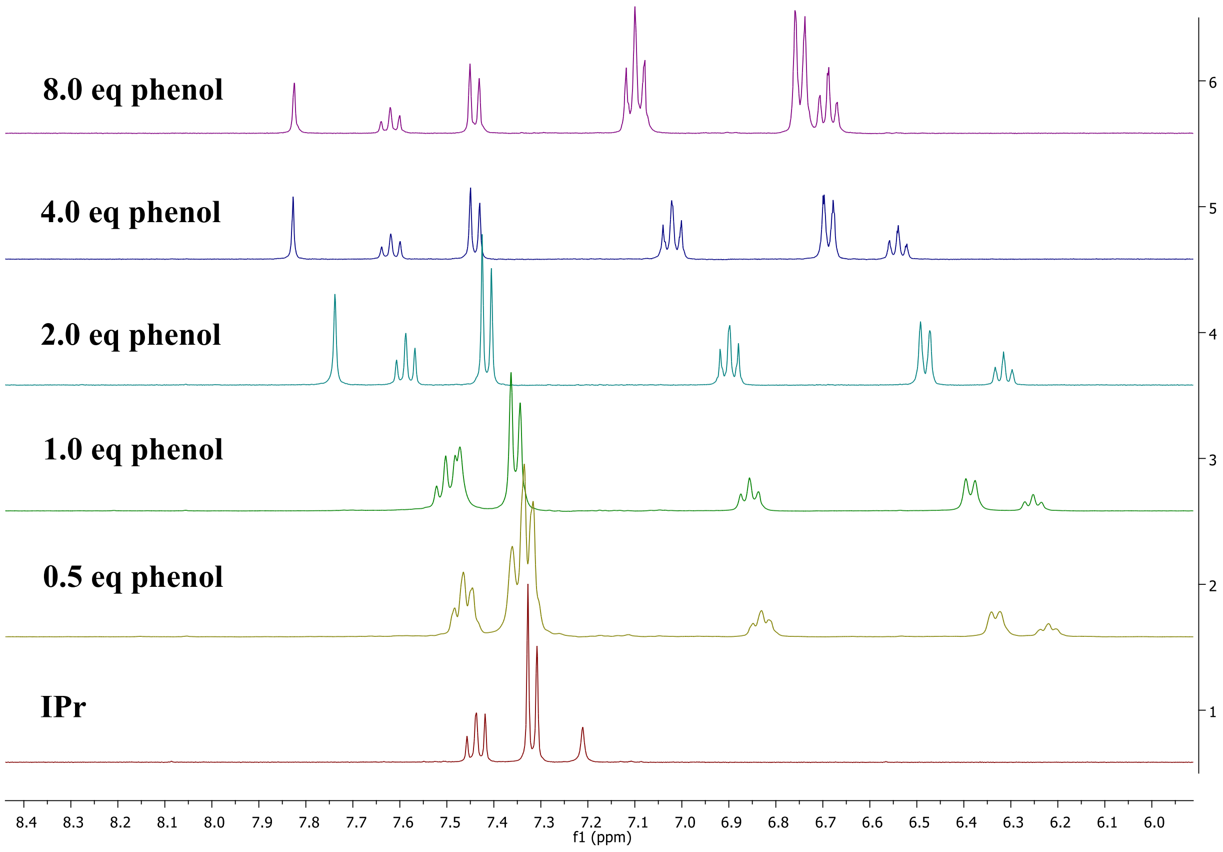

Figure S31. ^{1}H NMR spectra (400MHz, CD_3CN, 25 °C) of IPr with increasing concentrations of phenol.

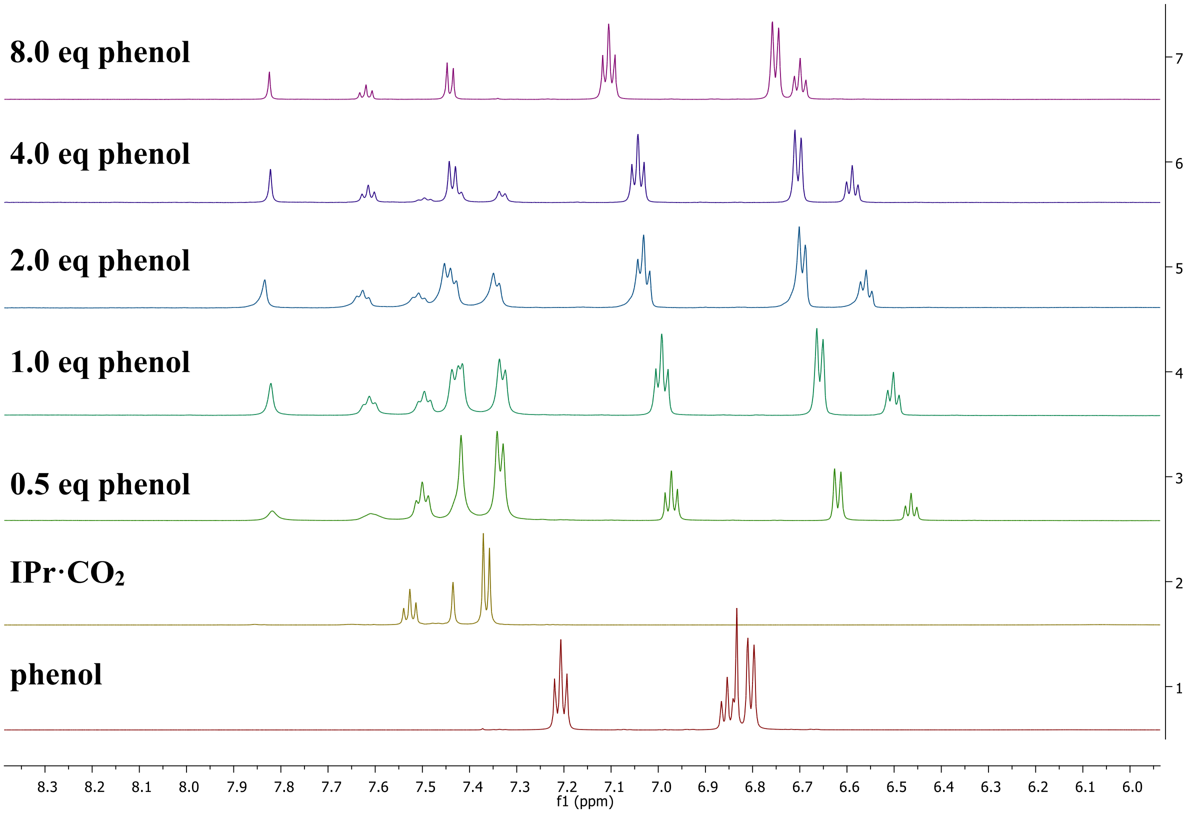

Figure S32. ^{1}H NMR spectra (600 MHz, CD_3CN, 25 °C) of phenol (bottom trace), IPr·CO_2 (second from bottom trace), and with increasing equivalents of phenol to IPr·CO_2 as labeled.

Kinetic Experiments

NMR Spectroscopy Kinetics Studies with [Pt(dmpe)$_2$](PF$_6$)$_2$ (1) and IPr·CO$_2$. The rate of hydride transfer was found for three concentrations of IPrCO$_2$. 1 mM [Pt(dmpe)$_2$](PF$_6$)$_2$ (**1**), 10-40 mM IPr·CO$_2$, and triphenylphosphine oxide (ca. 2 mg) were dissolved in CH$_3$CN. An aliquot was taken for the NMR tube and spectra were acquired within 15 minutes of making the solution. The integration of the triphenylphosphine oxide was used as a relative standard to monitor the decay of the hydride species. The natural logarithm of the [HPt(dmpe)$_2$](PF$_6$) (**3**) integration was plotted as a function of time in seconds to give a linear relationship. The slope of this first order relationship represents the rate of decay (k_{obs}) for that concentration of IPr·CO$_2$ (Table S2). The various rates were plotted against the concentration of IPr·CO$_2$ to yield the second order rate constant of 1.8 x 10^{-3} M^{-1} s^{-1} (rate law = k[HPt(dmpe)$_2$(PF$_6$)$_2$][IPr·CO$_2$]) (Figure S33).

Table S2. k_{obs} values for different concentrations of IPr·CO$_2$

[IPr·CO$_2$] (M)	k_{obs} (s^{-1})
0.01	1.03x10^{-5}
0.02	3.80x10^{-5}
0.04	6.60x10^{-5}

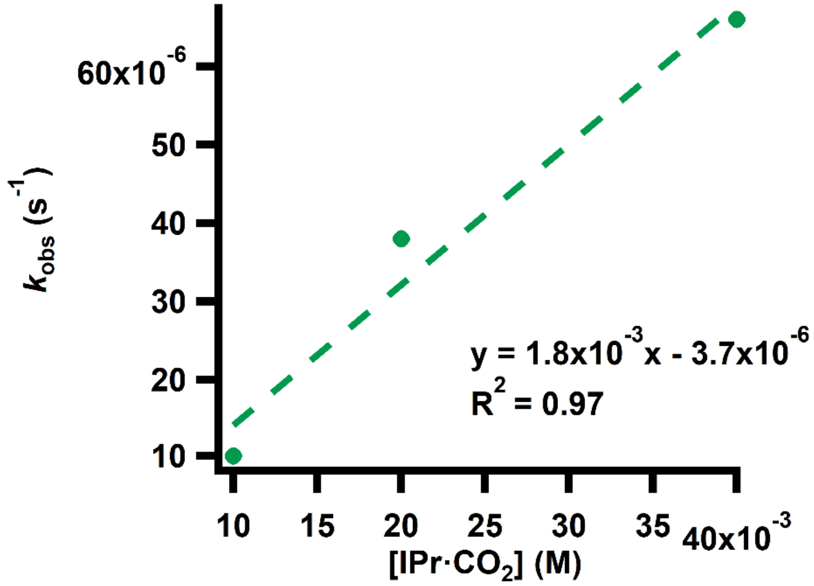

Figure S33. Determination of the second order rate constant for hydride transfer to IPr·CO$_2$. Multiple concentrations of IPr·CO$_2$ were used to give a second order rate constant of 1.8 x 10^{-3} M^{-1} s^{-1}.

UV-visible Spectroscopy Kinetics Studies with CO_2. The rate of hydride transfer to CO_2 was studied by UV-vis spectroscopy. In a cuvette, 2.9 mL of CH_3CN was sparged with a saturated mixed gas stream of CO_2 and N_2. The spectrometer was already set to start scanning (every 7 seconds) before adding 0.1 mL of [HPt(dmpe)$_2$](PF$_6$) (30 mM in CH_3CN) to the cuvette and inverting once to mix the solution. The percentages of CO_2 used were 15%, 30%, 45%, and 60% and balanced with N_2.

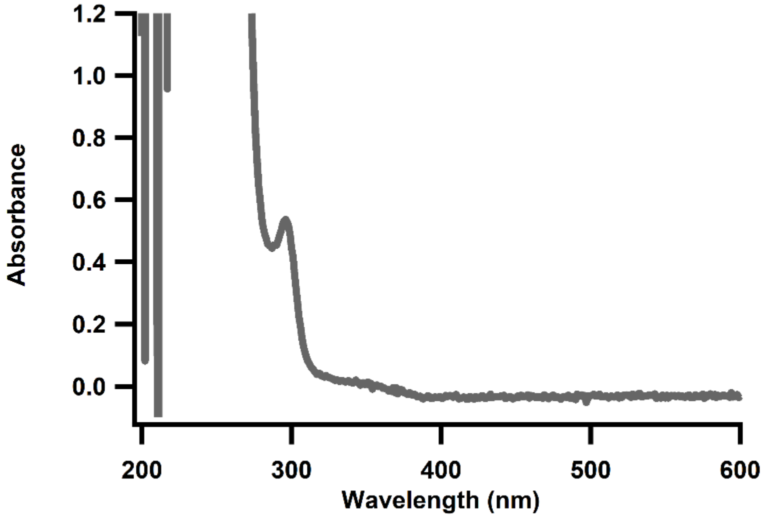

Figure S34. UV-vis spectrum of [Pt(dmpe)$_2$](PF$_6$)$_2$ (**1**) in CH_3CN with a max absorbance at 296 nm.

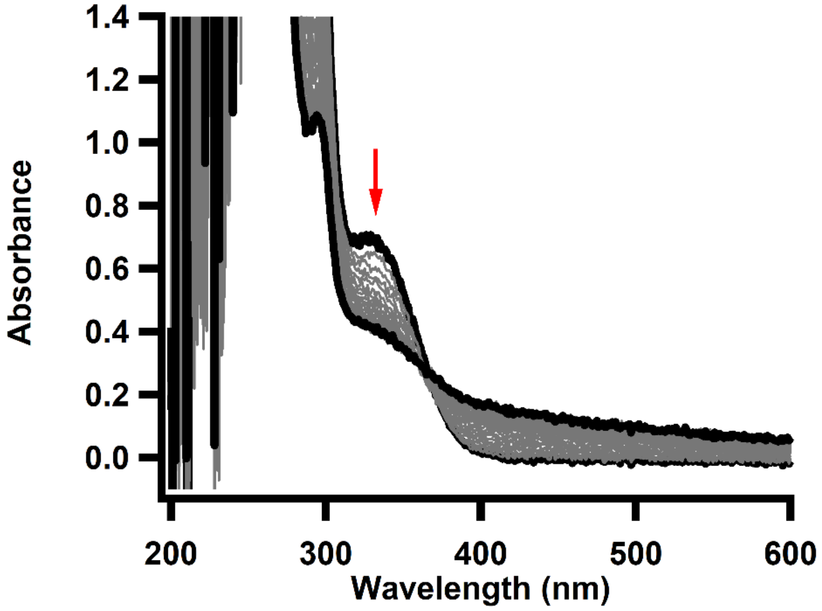

Figure S35. UV-vis decay spectra of [HPt(dmpe)$_2$](PF$_6$) (**3**) (1 mM) in CH_3CN sparged with 15% CO_2. Scans were collected every 7 seconds. [HPt(dmpe)$_2$](PF$_6$) (**3**) (λ_{max} = 332 nm) decays over the course of 98 seconds and the formation of [Pt(dmpe)$_2$](PF$_6$)$_2$ (**1**) is observed at 296 nm.

Table S3. Percentages and concentrations of CO_2 used for determining the second order rate constant from UV-vis spectroscopy.

**% CO_2**	**[CO_2] (M)**
4.5	0.0126
15	0.042
30	0.084
45	0.126

Table S4. Average k_{obs} values determined for hydride transfer to CO_2 at various concentrations of CO_2.

**[CO_2] (M)**	**k_{obs} (s^{-1})**
0.0126	0.007(2)
0.042	0.015(5)
0.084	0.024(5)
0.126	0.031(7)

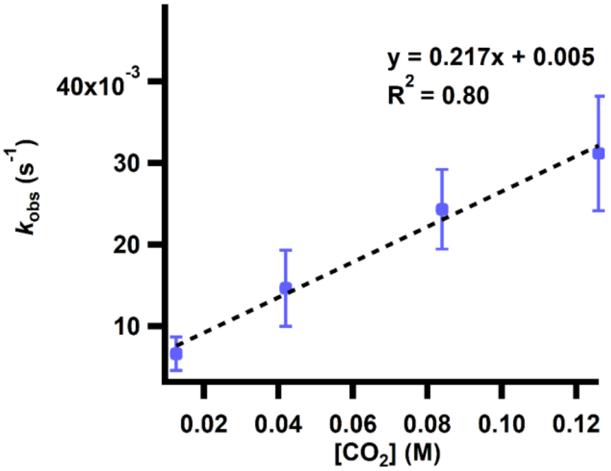

Figure S36. Determination of the second order rate constant of hydride transfer to CO_2. Multiple concentrations of CO_2 were used to give a rate constant of 0.22 M^{-1} s^{-1}.

UV-visible Spectroscopy Kinetic Study with IPr·CO_2 and phenol. The rate of CO_2 release form IPr·CO_2 was studied by UV-vis spectroscopy. IPr·CO_2 (15 mM) was dissolved in 3 mL of CH_3CN. The spectrometer was already set to start scanning (every 7 seconds) before the equimolar phenol (15 mM) was added.

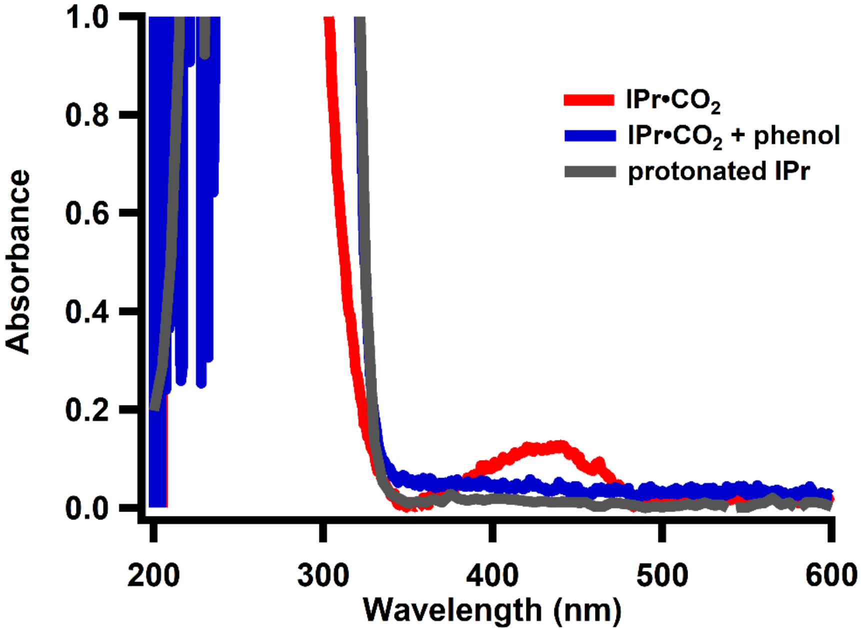

Figure S37. UV-vis spectrum of IPr·CO_2 (15 mM) (red) and after phenol (15 mM) was added (blue). A trace of protonated IPr is shown in gray.

Crystal Structure Data

X-ray Data Collection, Structure Solution and Refinement

A colorless crystal of approximate dimensions 0.152 x 0.164 x 0.267 mm was mounted in a cryoloop and transferred to a Bruker D8 Venture Photon III diffractometer system. The APEX5[9] program package was used to determine the unit-cell parameters and for data collection (3 sec/frame scan time). The raw frame data was processed using SAINT[10] and SADABS[11] to yield the reflection data file. Subsequent calculations were carried out using the SHELXTL[12]program package. The diffraction symmetry was *mmm* and the systematic absences were consistent with the orthorhombic space group $P2_12_12_1$ that was later determined to be correct.

The structure was solved by direct methods and refined on F^2 by full-matrix least-squares techniques. The analytical scattering factors[13] for neutral atoms were used throughout the analysis. Hydrogen atoms were located from a difference-Fourier map and refined (x,y,z and U_{iso}), except for H13, H13A, H14A to H14F, H15A to H15F, H22, H22A, H23A to H23F, and H24A to H24F, which were included using a riding model. C14, C15, C23, and C24 were disordered and included using multiple components, partial site occupancy factors, and restraints.

Least squares analysis yielded wR2 = 0.0791 and Goof = 1.037 for 655 variables refined against 8284 data (0.79 Å), R1 = 0.0313 for those 8054 data with I > 2.0s(I).

Table S5. Crystal data and structure refinement for the reaction of IPr and phenol.

Empirical formula	$C_{27}H_{37}N_2$•C_6H_5O•2(C_6H_5OH)	
Formula weight	670.90	
Temperature	100(2) K	
Wavelength	1.54178 Å	
Crystal system	Orthorhombic	
Space group	*$P2_12_12_1$*	
Unit cell dimensions	a = 12.8380(2) Å	α = 90°.
	b = 16.2192(3) Å	β = 90°.
	c = 18.8797(3) Å	γ = 90°.
Volume	3931.17(11) $Å^3$	
Z	4	
Density (calculated)	1.134 Mg/m^3	
Absorption coefficient	0.543 mm^{-1}	
F(000)	1448	
Crystal color	colourless	
Crystal size	0.267 x 0.164 x 0.152 mm^3	
Theta range for data collection	4.684 to 77.461°	
Index ranges	-16 <= *h* <= 16, -20 <= *k* <= 18, -23 <= *l* <= 23	
Reflections collected	52960	
Independent reflections	8284 [R(int) = 0.0325]	
Completeness to theta = 67.679°	99.6 %	
Absorption correction	Semi-empirical from equivalents	
Max. and min. transmission	0.8646 and 0.8151	
Refinement method	Full-matrix least-squares on F^2	
Data / restraints / parameters	8284 / 22 / 655	
Goodness-of-fit on F^2	1.037	
Final R indices [I>2sigma(I) = 8054 data]	R1 = 0.0313, wR2 = 0.0782	
R indices (all data, 0.79 Å)	R1 = 0.0323, wR2 = 0.0791	

Absolute structure parameter	-0.01(4)
Largest diff. peak and hole	0.167 and -0.192 e.Å^{-3}

Table S6. Hydrogen bonds for the reaction of IPr and phenol [Å and °].

D-H...A	d(D-H)	d(H...A)	d(D...A)	<(DHA)
O(2)-H(2A)...O(1)	0.93(3)	1.62(3)	2.5499(16)	174(3)
O(3)-H(3A)...O(1)	0.89(3)	1.69(3)	2.5792(17)	176(3)
C(1)-H(1)...O(1)	0.897(19)	2.130(19)	3.0252(18)	175.2(16)

Computational Details

Free energies for solution phase molecules (Figures 4 and 5) were calculated using ORCA 5.0.4.[14,15] Free energies (in the 1 M standard state) are composed according to:

$$G = G_{solv} + E_{ZPE} + H_{vib} + H_{trans} + H_{rot} + PV - T*(S_{vib} + S_{elec} + S_{trans} + S_{rot} - k_B\ln(24.5/1))$$

The solvated electronic energy was taken from an optimization performed with: the SMD[16] solvation model (CH_3CN); the TPSS0[17–19] functional with D3 dispersion corrections[20,21] (with Becke-Jones damping); the ma-TZVP(-f) basis[22,23] set and ECP.[24] The zero point energy and temperature-dependent terms (comprising standard statistical mechanical formula and a concentration correction from 1 atm to 1 M) were calculated from a Hessian resulting from a separate optimization performed in vacuum with the BP86 functional[25,26] with D3BJ dispersion corrections, the def2-SVP basis[23] and def2/J auxiliary basis. To alleviate the near linear dependence of diffuse functions on the many carbon atoms, we used only diffuse *p*-functions with exponent 0.05 on carbon. T = 298 K.[27]

The free energy of a proton at pH 29 in CH_3CN is based on the ideal 1 atm free energy of a proton, Tissandier et al.'s proton solvation energy in water,[28] Appel, et al.'s transfer free energy[29] from water to acetonitrile, and the pK_a of phenol. The proton free energy employed (-295.7 kcal/mol relative to a proton at rest in vacuum) is derived as:

DG(H^+: 1 atm) = H – TS = 1.48 kcal/mol – (298K)(26.04 eu) = -6.28 kcal/mol,

DG(H^+: 1atm → 1M aq) = -264 kcal/mol,[28]

DG(H^+: 1M,aq → 1M, CH_3CN) = 14 kcal/mol,[29]

DG(pH 0 → 29) = -1.36*29 = 39.4 kcal/mol.

References

(1) Miedaner, A.; Dubois, D. L.; Curtis, C. J.; Haltiwanger, R. C. Generation of Metal Formyl Complexes Using Nickel and Platinum Hydrides as Reducing Agents. *Organometallics*. **1993**, *12* (2) 299-303. https://doi.org/10.1021/om00026a014.

(2) Berning, D. E.; Noll, B. C.; DuBois, D. L. Relative Hydride, Proton, and Hydrogen Atom Transfer Abilities of [HM(Diphosphine)$_2$]PF$_6$ Complexes (M = Pt, Ni). *J Am Chem. Soc.* **1999**, *121* (49), 11432–11447. https://doi.org/10.1021/ja991888y.

(3) Ceballos, B. M.; Yang, J. Y. Directing the Reactivity of Metal Hydrides for Selective CO2 Reduction. *Proc. Natl. Acad. Sci. USA* **2018**, *115* (50), 12686–12691. https://doi.org/10.1073/pnas.1811396115.

(4) Sandford, C.; Edwards, M. A.; Klunder, K. J.; Hickey, D. P.; Li, M.; Barman, K.; Sigman, M. S.; White, H. S.; Minteer, S. D. A Synthetic Chemist's Guide to Electroanalytical Tools for Studying Reaction Mechanisms. *Chem. Sci.* **2019**, *10* (26), 6404–6422. https://doi.org/10.1039/C9SC01545K.

(5) Cunningham, D. W.; Yang, J. Y. Kinetic and Mechanistic Analysis of a Synthetic Reversible CO2/HCO2− Electrocatalyst. *Chem. Commun.* **2020**, *56* (85), 12965–12968. https://doi.org/10.1039/D0CC05556E.

(6) Savéant, J. *Elements of Molecular and Biomolecular Electrochemistry*; Wiley, 2006. https://doi.org/10.1002/0471758078.

(7) Saveant, J. M. *Investigation of Rates and Mechanisms of Reactions*; 1986.

(8) Ceballos, B. M.; Yang, J. Y. Highly Selective Electrocatalytic CO2 Reduction by [Pt(Dmpe)$_2$]$^{2+}$ through Kinetic and Thermodynamic Control. *Organometallics* **2020**, *39* (9), 1491–1496. https://doi.org/10.1021/acs.organomet.9b00720.

(9) APEX5 Version 2023.9-2, Bruker AXS, Inc.; Madison, WI 2023.

(10) SAINT Version 8.40b, Bruker AXS, Inc.; Madison, WI 2013.

(11) Sheldrick, G. M. SADABS, Version 2016/2, Bruker AXS, Inc.; Madison, WI 2016.

(12) Sheldrick, G. M. SHELXTL, Version 2019/1, Bruker AXS, Inc.; Madison, WI 2019.

(13) International Tables for Crystallography 1992, Vol. C., Dordrecht: Kluwer Academic Publishers.

(14) Neese, F. The ORCA Program System. *WIREs Computational Molecular Science* **2012**, *2* (1), 73–78. https://doi.org/10.1002/wcms.81.

(15) Neese, F. Software Update: The ORCA Program System—Version 5.0. *WIREs Computational Molecular Science* **2022**, *12* (5). https://doi.org/10.1002/wcms.1606.

(16) Marenich, A. V.; Cramer, C. J.; Truhlar, D. G. Universal Solvation Model Based on Solute Electron Density and on a Continuum Model of the Solvent Defined by the Bulk Dielectric Constant and Atomic Surface Tensions. *J. Phys. Chem. B* **2009**, *113* (18), 6378–6396. https://doi.org/10.1021/jp810292n.

(17) Tao, J.; Perdew, J. P.; Staroverov, V. N.; Scuseria, G. E. Climbing the Density Functional Ladder: Nonempirical Meta–Generalized Gradient Approximation Designed for Molecules and Solids. *Phys. Rev. Lett.* **2003**, *91* (14), 146401. https://doi.org/10.1103/PhysRevLett.91.146401.

(18) Perdew, J. P.; Tao, J.; Staroverov, V. N.; Scuseria, G. E. Meta-Generalized Gradient Approximation: Explanation of a Realistic Nonempirical Density Functional. *J. Chem. Phys.* **2004**, *120* (15), 6898–6911. https://doi.org/10.1063/1.1665298.

(19) Grimme, S. Accurate Calculation of the Heats of Formation for Large Main Group Compounds with Spin-Component Scaled MP2 Methods. *J. Phys. Chem. A* **2005**, *109* (13), 3067–3077. https://doi.org/10.1021/jp050036j.

(20) Grimme, S.; Antony, J.; Ehrlich, S.; Krieg, H. A Consistent and Accurate Ab Initio Parametrization of Density Functional Dispersion Correction (DFT-D) for the 94 Elements H-Pu. *J. Chem. Phys.* **2010**, *132* (15). https://doi.org/10.1063/1.3382344.

(21) Grimme, S.; Ehrlich, S.; Goerigk, L. Effect of the Damping Function in Dispersion Corrected Density Functional Theory. *J. Comput. Chem.* **2011**, *32* (7), 1456–1465. https://doi.org/10.1002/jcc.21759.

(22) Zheng, J.; Xu, X.; Truhlar, D. G. Minimally Augmented Karlsruhe Basis Sets. *Theor. Chem. Acc.* **2011**, *128* (3), 295–305. https://doi.org/10.1007/s00214-010-0846-z.

(23) Weigend, F.; Ahlrichs, R. Balanced Basis Sets of Split Valence, Triple Zeta Valence and Quadruple Zeta Valence Quality for H to Rn: Design and Assessment of Accuracy. *Phys. Chem. Chem. Phys.* **2005**, *7* (18), 3297. https://doi.org/10.1039/b508541a.

(24) Andrae, D.; Haeussermann, U.; Dolg, M.; Stoll, H.; Preuss, H. Energy-Adjusted Ab Initio Pseudopotentials for the Second and Third Row Transition Elements. *Theor. Chim. Acta.* **1990**, *77* (2), 123–141. https://doi.org/10.1007/BF01114537.

(25) Becke, A. D. Density-Functional Exchange-Energy Approximation with Correct Asymptotic Behavior. *Phys. Rev. A (Coll Park)* **1988**, *38* (6), 3098–3100. https://doi.org/10.1103/PhysRevA.38.3098.

(26) Perdew, J. P. Density-Functional Approximation for the Correlation Energy of the Inhomogeneous Electron Gas. *Phys. Rev. B* **1986**, *33* (12), 8822–8824. https://doi.org/10.1103/PhysRevB.33.8822.

(27) Weigend, F. Accurate Coulomb-Fitting Basis Sets for H to Rn. *Phys. Chem. Chem. Phys.* **2006**, *8* (9), 1057. https://doi.org/10.1039/b515623h.

(28) Tissandier, M. D.; Cowen, K. A.; Feng, W. Y.; Gundlach, E.; Cohen, M. H.; Earhart, A. D.; Coe, J. V.; Tuttle, T. R. The Proton's Absolute Aqueous Enthalpy and Gibbs Free Energy of Solvation from Cluster-Ion Solvation Data. *J. Phys. Chem. A* **1998**, *102* (40), 7787–7794. https://doi.org/10.1021/jp982638r.

(29) Pegis, M. L.; Roberts, J. A. S.; Wasylenko, D. J.; Mader, E. A.; Appel, A. M.; Mayer, J. M. Standard Reduction Potentials for Oxygen and Carbon Dioxide Couples in Acetonitrile and N, N-Dimethylformamide. *Inorg. Chem.* **2015**, *54* (24), 11883–11888. https://doi.org/10.1021/acs.inorgchem.5b02136.
